# Declination of Treatment, Racial and Ethnic Disparity, and Overall Survival in US Patients With Breast Cancer

**DOI:** 10.1001/jamanetworkopen.2024.9449

**Published:** 2024-05-09

**Authors:** Jincong Q. Freeman, James L. Li, Susan G. Fisher, Katharine A. Yao, Sean P. David, Dezheng Huo

**Affiliations:** 1 Department of Public Health Sciences, University of Chicago, Chicago, Illinois; 2 Center for Health and the Social Sciences, University of Chicago, Chicago, Illinois; 3 Pritzker School of Medicine, University of Chicago, Chicago, Illinois; 4 NorthShore Research Institute, NorthShore University HealthSystem, Evanston, Illinois; 5 Department of Surgery, NorthShore University HealthSystem, Evanston, Illinois; 6 Center for Clinical Cancer Genetics and Global Health, University of Chicago, Chicago, Illinois

## Abstract

**Importance:**

Declining treatment negatively affects health outcomes among patients with cancer. Limited research has investigated national trends of and factors associated with treatment declination or its association with overall survival (OS) among patients with breast cancer.

**Objectives:**

To examine trends and racial and ethnic disparities in treatment declination and racial and ethnic OS differences stratified by treatment decision in US patients with breast cancer.

**Design, Setting, and Participants:**

This retrospective cross-sectional study used data for patients with breast cancer from the 2004 to 2020 National Cancer Database. Four treatment modalities were assessed: chemotherapy, hormone therapy (HT), radiotherapy, and surgery. The chemotherapy cohort included patients with stage I to IV disease. The HT cohort included patients with stage I to IV hormone receptor–positive disease. The radiotherapy and surgery cohorts included patients with stage I to III disease. Data were analyzed from March to November 2023.

**Exposure:**

Race and ethnicity and other sociodemographic and clinicopathologic characteristics.

**Main Outcomes and Measures:**

Treatment decision, categorized as received or declined, was modeled using logistic regression. OS was modeled using Cox regression. Models were controlled for year of initial diagnosis, age, sex, health insurance, median household income, facility type, Charlson-Deyo comorbidity score, histology, American Joint Committee on Cancer stage, molecular subtype, and tumor grade.

**Results:**

The study included 2 837 446 patients (mean [SD] age, 61.6 [13.4] years; 99.1% female), with 1.7% American Indian, Alaska Native, or other patients; 3.5% Asian or Pacific Islander patients; 11.2% Black patients; 5.6% Hispanic patients; and 78.0% White patients. Of 1 296 488 patients who were offered chemotherapy, 124 721 (9.6%) declined; 99 276 of 1 635 916 patients (6.1%) declined radiotherapy; 94 363 of 1 893 339 patients (5.0%) declined HT; and 15 846 of 2 590 963 patients (0.6%) declined surgery. Compared with White patients, American Indian, Alaska Native, or other patients (adjusted odds ratio [AOR], 1.47; 95% CI, 1.26-1.72), Asian or Pacific Islander patients (AOR, 1.29; 95% CI, 1.15-1.44), and Black patients (AOR, 2.01; 95% CI, 1.89-2.14) were more likely to decline surgery; American Indian, Alaska Native, or other patients (AOR, 1.13; 95% CI, 1.05-1.21) and Asian or Pacific Islander patients (AOR, 1.21; 95% CI, 1.16-1.27) were more likely to decline chemotherapy; and Black patients were more likely to decline radiotherapy (AOR, 1.05; 95% CI, 1.02-1.08). Asian or Pacific Islander patients (AOR, 0.81; 95% CI, 0.77-0.85), Black patients (AOR, 0.86; 95% CI, 0.83-0.89), and Hispanic patients (AOR, 0.66; 95% CI, 0.63-0.69) were less likely to decline HT. Furthermore, Black patients who declined chemotherapy had a higher mortality risk than White patients (adjusted hazard ratio [AHR], 1.07; 95% CI, 1.02-1.13), while there were no OS differences between Black and White patients who declined HT (AHR, 1.05; 95% CI, 0.97-1.13) or radiotherapy (AHR, 0.98; 95% CI, 0.92-1.04).

**Conclusions and Relevance:**

This cross-sectional study highlights racial and ethnic disparities in treatment declination and OS, suggesting the need for equity-focused interventions, such as patient education on treatment benefits and improved patient-clinician communication and shared decision-making, to reduce disparities and improve patient survival.

## Key Points

**Question:**

What are the treatment declination trends and are there racial and ethnic disparities in treatment declination and overall survival among patients with breast cancer?

**Findings:**

In this cross-sectional study of 2 837 446 patients with breast cancer, the treatment declination rate was highest for chemotherapy and lowest for surgery. American Indian, Alaska Native, or other; Asian or Pacific Islander; and Black patients were more likely to decline chemotherapy, radiotherapy, or surgery than White patients; Asian or Pacific Islander, Black, and Hispanic patients were less likely to decline hormone therapy than White patients, with racial and ethnic disparities in overall survival differing by treatment decision.

**Meaning:**

These findings highlight racial and ethnic disparities in treatment declination and overall survival of patients with breast cancer, suggesting that equity-focused interventions are needed to address the disparities to improve patients’ survival.

## Teaser

This cross-sectional study among US patients with breast cancer assesses trends and racial and ethnic disparities in decisions to decline treatment and overall survival among those who declined treatment.

## Introduction

In the US, breast cancer (BC) is the most common malignant neoplasm and the second leading cause of cancer deaths among women, with an estimated 287 850 new diagnoses and 43 250 deaths in 2022.^1,2^ BC diagnosis and treatment can take a heavy toll on patients’ physical, mental, psychosocial, and financial health. Cancer treatment and care services require interdisciplinary and multidisciplinary collaborations and effective patient-clinician communication and shared decision-making, while respecting patient autonomy.^3^ Some patients with cancer choose to decline treatment despite clinician recommendations and treatment benefits. Declining curative treatment can have a detrimental effect on these patients’ short-term and long-term health outcomes and quality of life.^4,5,6^ Studies have documented elevated risks of all-cause and disease-specific mortality in patients with cancer who forgo treatment recommended by their clinicians.^7,8,9,10,11^

Previous research in colorectal,^8,9,11^ ovarian,^12^ lung,^13,14,15^ or mixed cancer cohorts^16,17^ has found that older age, racial and ethnic minority background (eg, Hispanic, non-Hispanic Asian or Pacific Islander, or non-Hispanic Black), low socioeconomic status, and late-stage presentations are associated with declination of therapies. For BC, several analyses have reported similar sociodemographic and clinical factors associated with treatment declination in this patient population.^7,10,18,19,20,21,22^ However, these studies focused on either chemotherapy or surgery only or the associations of treatment decisions with mortality, and most of them did not evaluate the pattern and long-term trends associated with treatment declination among patients with BC. Although a few studies have assessed racial and ethnic disparities in declination of surgery or chemotherapy, they largely focused on Black and Hispanic patients, and, to a lesser extent, Asian patients,^10,18,19,21^ and 2 analyses included only White women.^4,7^ There remain gaps in the literature regarding national trends in declination of treatment recommendations, racial and ethnic disparities, sociodemographic and clinicopathologic characteristics associated with treatment declination, and the implication of treatment declination for overall survival (OS) among patients with BC.

To fill these gaps, we conducted this study with the primary aim of examining trends and factors associated with declination of 4 treatment modalities (ie, chemotherapy, hormone therapy [HT], radiotherapy, and surgery), using a US nationwide oncology registry. The secondary aim was to assess the OS of patients with BC stratified by race and ethnicity and treatment decision.

## Methods

This cross-sectional study was granted a waiver for informed consent and a review exemption by the University of Chicago institutional review board because we used deidentified data that do not identify hospitals, health care practitioners, or patients. The study followed the Strengthening the Reporting of Observational Studies in Epidemiology (STROBE) reporting guideline.

### Data Source and Study Cohorts

This retrospective study analyzed data collected from patients with BC in the 2004 to 2020 National Cancer Database (NCDB), a joint project of the Commission on Cancer of the American College of Surgeons and the American Cancer Society.^23^ The NCDB is a clinical oncology registry that captures approximately 72% of new cancer diagnoses from more than 1500 Commission on Cancer–accredited programs in the US annually.^24,25,26^

We constructed 4 patient cohorts with 4 treatment modalities: chemotherapy, HT, radiotherapy, and surgery. Stage group was based on the American Joint Committee on Cancer cancer staging. The chemotherapy cohort included patients with stage I to IV disease who were recommended for chemotherapy in the neoadjuvant or adjuvant setting. The HT cohort consisted of patients with stage I to IV hormone receptor–positive BC with recommended HT. The radiotherapy or surgery cohort was limited to patients with stage I to III disease, because neither treatment is the standard of care for stage IV BC.

### Measures

Decision on recommended treatment was classified as received or declined. Chemotherapy, HT, radiotherapy, or surgery administered as the first course of therapy was categorized as received. If the treatment was recommended by a patient’s clinician but declined by the patient, their family members, or guardians, it was categorized as declined. Trends in declination of therapies or surgeries from 2004 to 2020 were assessed. Moreover, to examine the pattern of treatment declination, we tabulated the number of therapies patients eligible were for (only 1, 2, 3, all) and the number of therapies declined by the patients (0, only 1, 2, 3, all).

OS was defined as an event or censored at the time of death from all causes or last known patient contact. The index time for OS was the date of initial diagnosis of BC. Per the NCDB, mortality information was not available for patients diagnosed in 2020 due to limited time of follow-up; therefore, these patients were excluded from survival analysis. Median follow-up time and 5-year and 10-year rates of OS were calculated stratified by race and ethnicity and treatment decision.

Race and ethnicity were self-reported, and racial and ethnic groups were categorized as American Indian, Alaska Native, or other (non-Hispanic), Asian or Pacific Islander (non-Hispanic), Black (non-Hispanic), Hispanic, and White (non-Hispanic). Other is a racial and ethnic group listed in the NCDB and represents patients who were classified as other by local cancer registries. The NCDB does not specifically define race and ethnicity classified into other. Additional patient characteristics included age at diagnosis, sex assigned at birth, type of health insurance (uninsured, private, Medicaid, Medicare, and other government or unknown), median household income quartile (<$40 227, $40 227-$50 353, $50 354-$63 332, and ≥$63 333), rural-urban residence, facility type, Charlson-Deyo comorbidity index (CCI; 0, 1, and ≥2), cancer stage group, histology, molecular subtype, tumor grade, and year of initial diagnosis.

### Statistical Analysis

Sociodemographic and clinicopathologic factors were compared between treatment administration and declination using Pearson χ^2^ tests for nominal data and *t* tests for continuous data. To examine time trends in treatment declination, we fit generalized linear models with the log link and binomial distribution. Multivariable logistic regression was used to model the odds of treatment declination as a function of race and ethnicity and other patient characteristics. We fit separate logistic regression models for the 4 cohorts and reported adjusted odds ratios (AORs) with 95% CIs. The Kaplan-Meier method was used to estimate medial survival time, 5-year and 10-year OS rates, and corresponding 95% CIs. Stratified by treatment decision, we assessed differential OS by race and ethnicity using log-rank tests, followed by modeling the risk of all-cause mortality using multivariable Cox proportional hazards regression. Adjusted hazard ratios (AHRs) and 95% CIs were calculated. The level of significance was set at *P* < .05 and hypothesis tests were 2-sided.

Per the NCDB, patients who did not receive recommended treatment and did not have a reason noted or with an unknown status of treatment administration in their medical records were categorized as missing. We observed that the rate of missing treatment decision varied over time (eTable 1 in Supplement 1) and patient characteristics differed by missing status (eTables 2-5 in Supplement 1). Therefore, we conducted a sensitivity analysis using inverse probability weighting (IPW) to examine the robustness of the results. The probability of missing each treatment was estimated using multivariable logistic regression in the 4 patient cohorts. All statistical analyses were performed using the Stata 17 software package (StataCorp) from March to November 2023.

## Results

### Patient Characteristics

The study included 2 837 446 patients (mean [SD] age, 61.6 [13.4] years; 99.1% female), of whom 1.7% were American Indian, Alaska Native, or other, 3.5% were Asian or Pacific Islander, 11.2% were Black, 5.6% were Hispanic, and 78.0% were White. By insurance status, 49.9% of patients had private insurance or managed care, 39.3% of patients had Medicare insurance, and 6.3% of patients had Medicaid insurance. Most patients (55.6%) had stage I disease, and nearly three-quarters of patients (74.0%) had hormone receptor-positive and *ERBB2*-negative disease (eTable 6 in Supplement 1).

### Prevalence of and Trends in Treatment Declination

**Figure 1.**
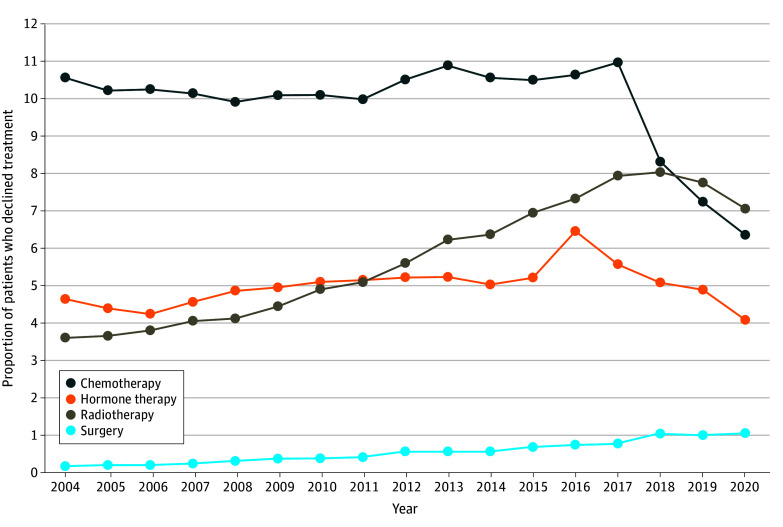
Adjusted Proportions for Declination of Recommended Treatment Among Patients With Breast Cancer Over Time

Overall, 124 721 of 1 296 488 patients (9.6%) who were offered chemotherapy declined; 99 276 of 1 635 916 patients (6.1%) declined radiotherapy; 94 363 of 1 893 339 patients (5.0%) declined HT; and 15 846 of 2 590 963 patients (0.6%) declined surgery. Regarding the pattern of declination, 8516 patients (0.4%) declined all treatments for which they were eligible and 240 223 patients (9.8%) declined 1 to 3 therapies; 2 210 675 (89.9) patients received all recommended treatments (Table 1). From 2004 to 2020, there were significant increasing trends in declination of HT (change per year, 1.97%; 95% CI, 0.50% to 3.45%; *P* for trend = .008), radiotherapy (change per year, 5.62%; 95% CI, 4.73% to 6.52%; *P* for trend < .001), and surgery (change per year, 11.12%; 95% CI, 8.43% to 13.88%; *P* for trend < .001), while the declination of chemotherapy decreased over time (change per year, –0.96%; 95% CI, –1.07% to –0.84%; *P* for trend < .001) (Figure 1). Because the IPW-adjusted (eTable 7 in Supplement 1) and IPW-unadjusted AORs (Table 2) were very similar, we report the AORs and 95% CIs without missingness adjustment.

**Table 1.** Patterns of Declination of Recommended Cancer Therapies in Patients With Breast Cancer

Recommended therapies declined, No.	Therapies recommended, No.			
	1	2	3	4
None (ie, received all recommended therapies)	188 140 (98.5)	552 402 (93.0)	1 018 420 (89.3)	451 713 (84.5)
1	2778 (1.5)	39 509 (6.7)	96 502 (8.5)	55 246 (10.3)
Any 2	NA	1908 (0.3)	23 451 (2.1)	14 962 (2.8)
Any 3 therapies	NA	NA	1754 (0.2)	10 553 (2.0)
All 4	NA	NA	NA	2076 (0.4)

Abbreviation: NA, not applicable.

**Table 2.** Sociodemographic and Clinicopathologic Factors Associated with Treatment Declination: Multivariable Logistic Regression

Variable	AOR (95% CI)			
	Chemotherapy cohort^a^^,^^b^	Hormone therapy cohort^c^^,^^d^	Radiotherapy cohort^e^^,^^b^	Surgery cohort^e^^,^^b^
Race and ethnicity				
American Indian, Alaska Native, or Other^f^	1.13 (1.05-1.21)	1.04 (0.96-1.11)	1.02 (0.94-1.09)	1.47 (1.26-1.72)
Asian or Pacific Islander	1.21 (1.16-1.27)	0.81 (0.77-0.85)	1.01 (0.96-1.06)	1.29 (1.15-1.44)
Black	1.03 (1.01-1.06)	0.86 (0.83-0.89)	1.05 (1.02-1.08)	2.01 (1.89-2.14)
Hispanic	0.78 (0.75-0.82)	0.66 (0.63-0.69)	0.74 (0.70-0.77)	0.80 (0.71-0.89)
White	1 [Reference]	1 [Reference]	1 [Reference]	1 [Reference]
Age at diagnosis, per 10-y increase	2.38 (2.35-2.40)	1.44 (1.42-1.45)	2.08 (2.06-2.10)	2.83 (2.77-2.90)
Sex				
Male	1 [Reference]	1 [Reference]	1 [Reference]	1 [Reference]
Female	1.34 (1.23-1.47)	1.35 (1.22-1.49)	1.04 (0.94-1.16)	2.02 (1.59-2.57)
Type of health insurance				
Uninsured	1.61 (1.51-1.72)	1.61 (1.49-1.73)	1.97 (1.83-2.12)	4.83 (4.22-5.51)
Private or managed care	1 [Reference]	1 [Reference]	1 [Reference]	1 [Reference]
Medicaid	1.51 (1.46-1.57)	1.44 (1.38-1.50)	1.87 (1.79-1.94)	3.19 (2.91-3.48)
Medicare	1.04 (1.01-1.06)	1.02 (0.99-1.04)	1.09 (1.07-1.12)	0.94 (0.89-1.00)
Other government or unknown	1.02 (0.96-1.09)	1.00 (0.93-1.07)	1.09 (1.02-1.16)	1.17 (1.00-1.38)
Median household income quartiles^g^				
<$40 227	1.04 (1.01-1.07)	0.87 (0.84-0.90)	1.16 (1.13-1.20)	1.14 (1.07-1.22)
$40 227–$50 353	1.06 (1.03-1.09)	0.95 (0.92-0.97)	1.11 (1.08-1.13)	1.13 (1.07-1.20)
$50 354–$63 332	1.05 (1.03-1.08)	1.00 (0.98-1.02)	1.09 (1.07-1.12)	1.06 (1.01-1.12)
≥$63 333	1 [Reference]	1 [Reference]	1 [Reference]	1 [Reference]
Rural/urban area^h^				
Metropolitan	1 [Reference]	1 [Reference]	1 [Reference]	1 [Reference]
Urban	0.94 (0.92-0.97)	0.96 (0.93-0.99)	1.01 (0.99-1.04)	0.83 (0.77-0.89)
Rural	0.92 (0.85-0.98)	0.96 (0.89-1.03)	1.05 (0.98-1.12)	0.68 (0.55-0.83)
Type of cancer program				
Community	0.94 (0.91-0.98)	1.07 (1.03-1.11)	0.92 (0.89-0.95)	0.89 (0.82-0.97)
Comprehensive community	1.10 (1.08-1.13)	1.19 (1.16-1.22)	1.00 (0.98-1.02)	0.88 (0.83-0.92)
Academic or research	1 [Reference]	1 [Reference]	1 [Reference]	1 [Reference]
Integrated network	1.03 (1.00-1.05)	1.13 (1.10-1.16)	1.03 (1.01-1.06)	0.90 (0.85-0.96)
Charlson-Deyo comorbidity index				
0	1 [Reference]	1 [Reference]	1 [Reference]	1 [Reference]
1	0.98 (0.95-1.00)	0.89 (0.87-0.91)	1.06 (1.03-1.08)	0.75 (0.70-0.80)
≥2	1.24 (1.20-1.29)	1.03 (0.99-1.07)	1.38 (1.34-1.43)	1.03 (0.96-1.11)
AJCC stage group				
I	1 [Reference]	1 [Reference]	1 [Reference]	1 [Reference]
II	0.49 (0.48-0.50)	0.73 (0.71-0.74)	1.26 (1.23-1.28)	2.69 (2.56-2.83)
III	0.26 (0.25-0.27)	0.59 (0.56-0.61)	1.20 (1.16-1.23)	4.32 (4.06-4.61)
IV	0.25 (0.24-0.26)	0.31 (0.29-0.34)	-	-
Tumor grade				
1	2.30 (2.24-2.37)	1.19 (1.16-1.22)	1.21 (1.17-1.24)	1.33 (1.24-1.43)
2	1.51 (1.48-1.54)	0.93 (0.91-0.96)	1.04 (1.01-1.06)	1.27 (1.20-1.34)
3	1 [Reference]	1 [Reference]	1 [Reference]	1 [Reference]

Abbreviations: AOR, adjusted odds ratio; AJCC, American Joint Committee on Cancer.

^a^ Among patients with stage I to IV breast cancer.

^b^ Additionally adjusted for histologic type, molecular subtype, and year of initial diagnosis.

^c^ Among patients with stage I to IV hormone receptor–positive breast cancer.

^d^ Additionally adjusted for histologic type, *ERBB2* status, and year of initial diagnosis.

^e^ Among patients with stage I to III breast cancer.

^f^ Other is a racial and ethnic group listed in the National Cancer Database and represents patients who were classified as Other by local cancer registries. The National Cancer Database does not specifically define race and ethnicity classified into other.

^g^ Based on the 2016 American Community Survey data, spanning years 2012 to 2016 and adjusted for 2016 inflation.

^h^ Measured by matching the state and county FIPS code of the patient recorded at the time of diagnosis against 2013 files published by the United States Department of Agriculture Economic Research Service.

### Racial and Ethnic Disparities and Factors Associated With Treatment Declination

In the chemotherapy cohort, 10.3% of White patients declined, compared with 8.7% of American Indian, Alaska Native, or other patients; 8.8% of Asian or Pacific Islander patients; 8.1% of Black patients; and 5.7% of Hispanic patients (*P* < .001) (eTable 8 in Supplement 1). After covariate adjustment, American Indian, Alaska Native, or other patients (AOR, 1.13; 95% CI, 1.05 to 1.21), Asian or Pacific Islander patients (AOR, 1.21; 95% CI, 1.16-1.27), and Black patients (AOR, 1.03; 95% CI, 1.01 to 1.06) were more likely to decline chemotherapy, while Hispanic patients (AOR, 0.78; 95%, 0.75 to 0.82) were less likely than White patients to decline chemotherapy (Table 2). Older age was associated with greater odds of declination (AOR per 10-year increase, 2.38 ; 95% CI, 2.35 to 2.40). Compared with privately insured patients, uninsured patients (AOR, 1.61; 95% CI, 1.51 to 1.72) and patients with Medicaid (AOR, 1.51; 95% CI, 1.46 to 1.57) had greater odds of declination. Patients with a lower median household income or tumor grade had higher odds of declining chemotherapy, while those with late-stage disease were less likely to decline (Table 2). To explore chemotherapy decisions based on multigene assays, we performed a subgroup analysis of patients with early-stage, hormone receptor–positive and *ERBB2*-negative BC after surgery. Consistent in both 21-gene and 70-gene assay groups, patients with high risk scores were less likely to have declined chemotherapy than those with low to intermediate risk scores, while those who were not tested had a declination rate in the middle (eTable 9 in Supplement 1).

In the HT cohort, the distribution of treatment declination differed by race and ethnicity (American Indian, Alaska Native, or other, 4.8%; Asian or Pacific Islander, 4.1%; Black, 4.2%; Hispanic, 3%; and White, 5.2%; *P* < .001) (eTable 10 in Supplement 1). After controlling for potential confounders, American Indian, Alaska Native, or other patients (AOR, 0.66; 95% CI, 0.63 to 0.69), Asian or Pacific Islander patients (AOR, 0.81; 95% CI, 0.77 to 0.85), and Black patients (AOR, 0.86; 95% CI, 0.83 to 0.89) were less likely to decline HT than White patients (Table 2). Older age was associated with higher odds of declination (AOR per 10-year increase, 1.44; 95% CI, 1.42 to 1.45). Uninsured patients (AOR, 1.61; 95% CI, 1.49 to 1.73) and patients with Medicaid (AOR, 1.44; 95% CI, 1.38 to 1.50) had greater odds of declination than privately insured patients. Late-stage disease was associated with lower odds of declining HT (Table 2).

In the radiotherapy cohort, the treatment declination rates were 5.5% for American Indian, Alaska Native, or other patients, 5.2% for Asian or Pacific Islander patients, 6.2% for Black patients, 4.1% for Hispanic patients, and 6.2% for White patients (*P* < .001) (eTable 11 in Supplement 1). On multivariable analysis, Black patients (AOR, 1.05; 95% CI, 1.02 to 1.08) were more likely to decline radiotherapy, while Hispanic patients (AOR, 0.74; 95% CI, 0.70 to 0.77) were less likely to decline radiotherapy than White patients (Table 2). Older patients had greater odds of declination (AOR per 10-year increase, 2.08; 95% CI, 2.06 to 2.10). Compared with privately insured patients, uninsured patients (AOR, 1.97; 95% CI, 1.83 to 2.12), patients with Medicaid (AOR, 1.87; 95% CI, 1.79 to 1.94), and patients with Medicare (AOR, 1.09; 95% CI, 1.07 to 1.12) had a higher likelihood of declining treatment. Having a lower median household income, greater CCI scores, stage II to III disease, or grade 1 to 2 disease were associated with greater odds of declination (Table 2).

In the surgery cohort, 0.7% of American Indian, Alaska Native, or other patients, 0.6% of Asian or Pacific Islander patients, 1.1% of Black patients, 0.4% of Hispanic patients, and 0.6% of White patients declined (*P* < .001) (eTable 12 in Supplement 1). After adjusting for covariates, American Indian, Alaska Native, or other patients (AOR, 1.47; 95% CI, 1.26 to 1.72), Asian or Pacific Islander patients (AOR, 1.29; 95% CI, 1.15 to 1.44), and Black patients (AOR, 2.01; 95% CI, 1.89 to 2.14) were more likely to decline, while Hispanic patients (AOR, 0.80; 95% CI, 0.71 to 0.89) were less likely to decline surgery than White patients (Table 2). Older patients had greater odds of declination (AOR per 10-year increase, 2.83; 95% CI, 2.77 to 2.90). Patients without insurance (AOR, 4.83; 95% CI, 4.22 to 5.51) and patients with Medicaid (AOR, 3.19; 95% CI, 2.91 to 3.48) had higher odds of declining than privately insured patients. Having a median household income of less than $40 227 (AOR,1.14; 95% CI, 1.07 to 1.22), $40 227 to $50 353 (AOR, 1.13; 95% CI, 1.07 to 1.20), or $50 354 to $63 332 (AOR, 1.06; 95% CI, 1.01 to 1.12) was associated with higher odds of declining surgery. Patients with late-stage disease or lower tumor grade were more likely to decline surgery (Table 2).

### Racial and Ethnic OS Differences by Treatment Decision

**Figure 2.**
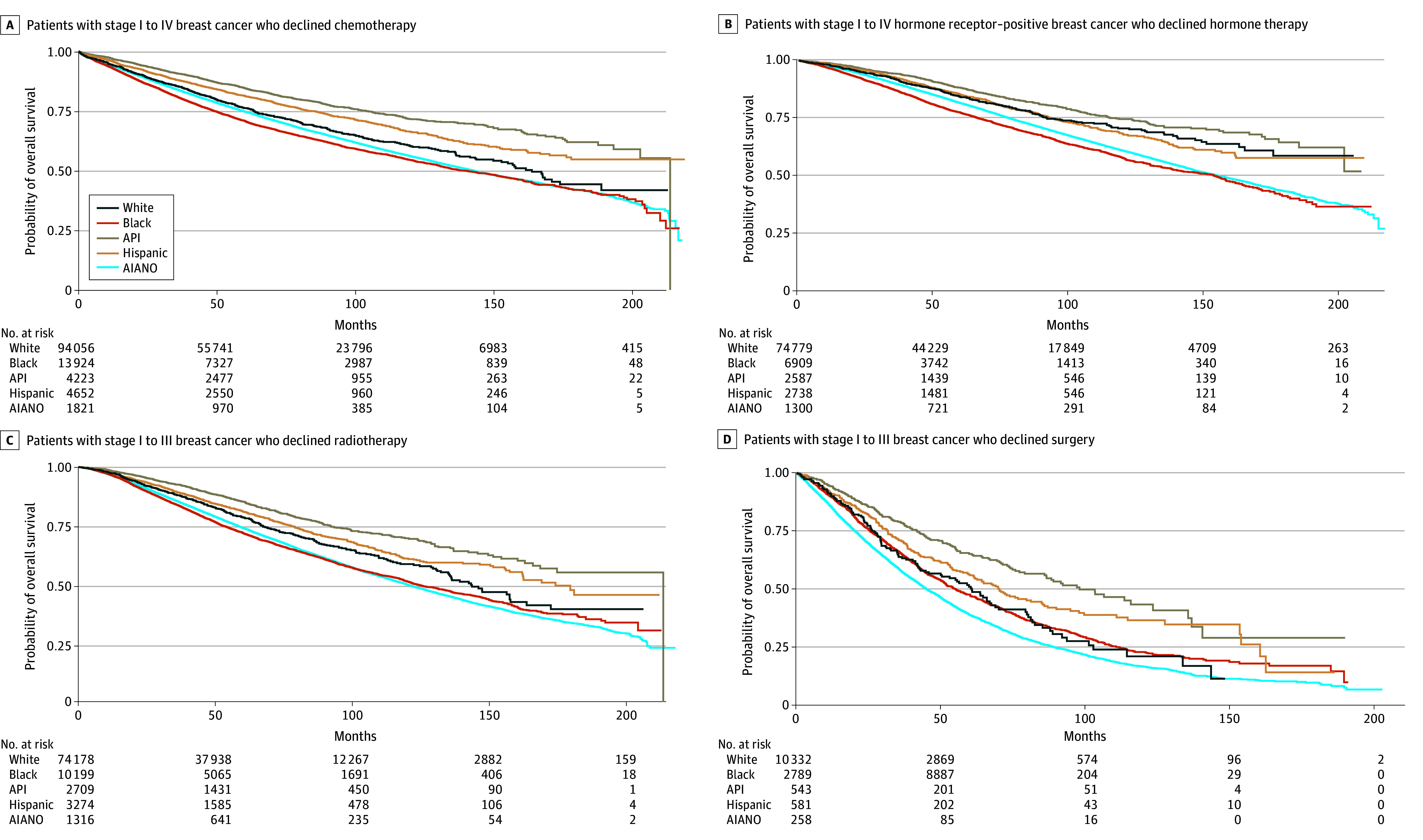
Kaplan-Meier Curves for Overall Survival Stratified by Race and Ethnicity in Patients Who Declined Treatment AIANO, American Indian, Alaska Native, or other; API, Asian or Pacific Islander. Other is a racial and ethnic group listed in the National Cancer Database and represents patients who were classified as other by local cancer registries. The National Cancer Database does not specifically define race and ethnicity classified into Other. Kaplan-Meier curves for patients who received treatment are provided in the eFigure in Supplement 1.

Consistent across all treatment cohorts, patients who received treatment had a longer median follow-up time (eTable 13 in Supplement 1) and higher 5-year and 10-year OS survival rates (eTable 14 in Supplement 1) than patients who declined treatment. When stratified by treatment decision, there were significant differences in OS across racial and ethnic groups (Figure 2 and eFigure in Supplement 1). In the adjusted Cox models (Table 3), Black patients who received chemotherapy (AHR, 1.15; 95% CI, 1.13 to 1.17), HT (AHR, 1.15; 95% CI, 1.13 to 1.17), radiotherapy (AHR, 1.13; 95% CI, 1.11 to 1.16), or surgery (AHR, 1.10; 95% CI, 1.09 to 1.12) had a greater risk of dying than White patients who received the treatment. Among patients who declined chemotherapy, Black patients also had a higher mortality risk than White patients (aHR, 1.07; 95% CI, 1.02 to 1.13) (Table 3). A similar OS rate was observed between Black and White patients who declined HT (AHR, 1.05; 95% CI, 0.97 to 1.13) or radiotherapy (AHR, 0.98; 95% CI, 0.92 to 1.04). Among patients who declined surgery, Black patients had a lower mortality risk than White patients (aHR, 0.82; 95% CI, 0.75 to 0.91). Regardless of treatment decision, American Indian, Alaska Native, or other; Asian or Pacific Islander; and Hispanic patients had a lower risk of dying than White patients (Table 3). Additionally, no insurance or public insurance, lower median household income, higher CCI scores, and late-stage disease were independently associated with a greater mortality risk among patients with BC stratified by treatment decision across all cohorts (eTables 15-18 in Supplement 1).

**Table 3.** Racial and Ethnic Differences in Overall Survival Among Patients With Breast Cancer Stratified by Treatment Decision

Race and ethnicity	HR (95% CI)					
	Among patients who declined treatment			Among patients who received treatment		
	Unadjusted	Model 1^a^	Model 2^b^	Unadjusted	Model 1^a^	Model 2^b^
**Chemotherapy cohort** ^c^						
American Indian, Alaska Native, or other^d^	0.90 (0.83-0.98)	1.05 (0.95-1.15)	0.86 (0.75-1.00)	0.85 (0.83-0.88)	0.89 (0.86-0.93)	0.88 (0.83-0.93)
Asian or Pacific Islander	0.56 (0.52-0.60)	0.73 (0.67-0.78)	0.68 (0.61-0.75)	0.65 (0.63-0.66)	0.74 (0.71-0.76)	0.73 (0.70-0.76)
Black	1.10 (1.07-1.14)	1.25 (1.20-1.29)	1.07 (1.02-1.13)	1.44 (1.43-1.46)	1.33 (1.32-1.35)	1.15 (1.13-1.17)
Hispanic	0.71 (0.66-0.75)	0.80 (0.75-0.86)	0.70 (0.63-0.77)	0.87 (0.85-0.89)	0.81 (0.79-0.83)	0.77 (0.75-0.80)
White	1 [Reference]	1 [Reference]	1 [Reference]	1 [Reference]	1 [Reference]	1 [Reference]
**Hormone therapy cohort** ^e^						
American Indian, Alaska Native, or other^d^	0.74 (0.65-0.84)	1.02 (0.89-1.17)	0.78 (0.64-0.95)	0.79 (0.77-0.82)	0.92 (0.88-0.95)	0.87 (0.82-0.91)
Asian or Pacific Islander	0.58 (0.53-0.65)	0.86 (0.77-0.96)	0.73 (0.63-0.84)	0.54 (0.53-0.56)	0.71 (0.69-0.73)	0.68 (0.66-0.71)
Black	1.16 (1.11-1.22)	1.41 (1.34-1.49)	1.05 (0.97-1.13)	1.29 (1.28-1.31)	1.30 (1.28-1.32)	1.15 (1.13-1.17)
Hispanic	0.76 (0.69-0.83)	0.95 (0.86-1.05)	0.81 (0.71-0.92)	0.73 (0.72-0.74)	0.80 (0.78-0.82)	0.75 (0.73-0.77)
White	1 [Reference]	1 [Reference]	1 [Reference]	1 [Reference]	1 [Reference]	1 [Reference]
**Radiotherapy cohort** ^f^						
American Indian, Alaska Native, or other^d^	0.81 (0.72-0.90)	1.04 (0.92-1.16)	0.95 (0.81-1.12)	0.79 (0.77-0.82)	0.92 (0.88-0.95)	0.88 (0.83-0.94)
Asian or Pacific Islander	0.54 (0.49-0.59)	0.74 (0.67-0.82)	0.71 (0.62-0.80)	0.60 (0.58-0.61)	0.75 (0.73-0.78)	0.70 (0.66-0.73)
Black	1.02 (0.99-1.06)	1.21 (1.16-1.26)	0.98 (0.92-1.04)	1.31 (1.29-1.33)	1.37 (1.35-1.39)	1.13 (1.11-1.16)
Hispanic	0.69 (0.64-0.75)	0.86 (0.79-0.93)	0.74 (0.66-0.82)	0.81 (0.79-0.82)	0.86 (0.84-0.88)	0.79 (0.76-0.81)
White	1 [Reference]	1 [Reference]	1 [Reference]	1 [Reference]	1 [Reference]	1 [Reference]
**Surgery cohort** ^f^						
American Indian, Alaska Native, or other^d^	0.79 (0.66-0.94)	0.87 (0.72-1.06)	0.80 (0.62-1.03)	0.75 (0.73-0.77)	0.90 (0.87-0.92)	0.87 (0.83-0.91)
Asian or Pacific Islander	0.45 (0.39-0.53)	0.57 (0.48-0.68)	0.56 (0.44-0.71)	0.53 (0.52-0.54)	0.71 (0.69-0.73)	0.68 (0.66-0.71)
Black	0.79 (0.74-0.83)	0.95 (0.88-1.01)	0.82 (0.75-0.91)	1.22 (1.21-1.23)	1.31 (1.30-1.33)	1.10 (1.09-1.12)
Hispanic	0.61 (0.53-0.69)	0.73 (0.64-0.85)	0.69 (0.57-0.84)	0.72 (0.71-0.74)	0.83 (0.81-0.84)	0.76 (0.74-0.78)
White	1 [Reference]	1 [Reference]	1 [Reference]	1 [Reference]	1 [Reference]	1 [Reference]

Abbreviation: HR, hazard ratio.

^a^ Adjusted for age at diagnosis, sex, type of health insurance, median household income quartile, and type of cancer program.

^b^ Adjusted for age at diagnosis, sex, type of health insurance, median household income quartile, type of cancer program, Charlson-Deyo comorbidity score, histology, American Joint Committee on Cancer stage group, molecular subtype (in the hormone therapy cohort: only ERBB2 status was adjusted for), tumor grade, and year of initial diagnosis.

^c^ Among patients with stage I-IV breast cancer.

^d^ Other is a racial and ethnic group listed in the National Cancer Database and represents patients who were classified as Other by local cancer registries. The National Cancer Database does not specifically define race and ethnicity classified into other.

^e^ Among patients with stage I to IV, hormone receptor–positive breast cancer.

^f^ Among patients with stage I to III breast cancer.

## Discussion

In this cross-sectional study using data from a large retrospective cohort of patients with BC, we found significant increasing trends in declination of HT, radiotherapy, and surgery from 2004 to 2020 and racial and ethnic and socioeconomic disparities in treatment declination. In particular, the increasing declination of treatment recommendations was more pronounced for radiotherapy and surgery. Older age, having public or no insurance, lower median household income, comorbidities, nonmetastatic disease, and lower tumor grade were associated with treatment declination. Furthermore, racial and ethnic differences in OS varied by treatment decision. Specifically, Black patients who declined chemotherapy had a greater mortality risk than White patients, while there were no OS differences between Black and White patients who declined HT or radiotherapy.

Our study expands on prior research findings by including radiotherapy and HT (in addition to chemotherapy and surgery), American Indian, Alaska Native, or other and Asian or Pacific Islander races and ethnicities as well as pattern and long-term trends of treatment declination. We found that 1 in 10 patients declined at least 1 type of recommended treatment, 1 in 10 patients declined chemotherapy, 5.0% to 6.0% of patients declined HT or radiotherapy, and less than 1.0% of patients declined surgery. These results are aligned with prior study observations in patients with BC using the Surveillance, Epidemiology, and End Results (SEER) and early-year NCDB data.^4,7,19,20,21,22^ Fwelo et al^21^ and Gaitanidis et al^10^ further observed increasing trends in 2004 to 2013 and 2010 to 2017 SEER data. However, these studies^10,21^ assessed surgery only; whereas we found that rates of declination of HT and radiotherapy also significantly increased, while the chemotherapy declination rate decreased from 2004 to 2020. Given that the exact reasons for declining treatment recommendations are not collected by the NCDB, it is unclear what has driven the increases or decrease over time. Meanwhile, it is important to note the decreased trends in declination of chemotherapy between 2018 and 2020, which we hypothesized were probably due to the more accurate chemotherapy decisions based on multigene assays, eg, the 21-gene assay and the 70-gene assay. The findings from our subgroup analysis suggest that multigene assay testing results probably influence the decision or receipt of chemotherapy and may partially explain the decreasing trend in the declination of chemotherapy recommendations from 2018 to 2020, as more patients received multigene assay testing in recent years. Future investigations are needed to decipher the growing trends and patterns of treatment declination in populations of patients with BC.

Compared with White patients, Black patients were more likely to decline chemotherapy or surgery; Hispanic patients had a 20% lower likelihood of declining either treatment. Our results support previous findings, as Rapp et al^17^ and Shahi et al^22^ have reported that Black patients were twice as likely as White patients to decline surgery in early-stage BC cohorts. Studies also have documented that among patients with stage III to IV BC or hormone receptor–positive and *ERBB2*-negative BC and high-risk scores on multigene assays, Black patients had a 9.0% to 20.0% greater likelihood of declining chemotherapy, while Hispanic patients were 18.0% less likely to do so, compared with White patients.^18,19^ However, these studies did not compare American Indian, Alaska Native, or other patients or Asian or Pacific Islander patients with White patients; whereas, we found that American Indian, Alaska Native, or other patients and Asian or Pacific Islander patients were 13.0% and 21.0% more likely to decline chemotherapy and were 29.0% and 47.0% more likely to decline surgery, respectively. Patients from racial and ethnic minority groups, except American Indian, Alaska Native, or other patients, were 19.0% to 34.0% less likely than White patients to decline HT. Black patients were 5.0% more likely to decline radiotherapy and Hispanic patients had a 26.0% lower likelihood of declining radiotherapy. In addition, older age, lack of insurance or Medicaid, lower median household income, advanced stage group, and higher tumor grade were associated with a significantly greater likelihood of declining systemic therapies or surgery, suggesting that differential rates of treatment declination not only are affected by clinicopathological factors but also may reflect socioeconomic disparities.

Qualitative studies have indicated that older patients with metastatic cancer or advanced chronic conditions forgo clinician recommendations because of diagnosis denial and fear of treatment adverse effects.^27,28,29,30^ Patient-clinician communication, shared decision-making, and trust can affect patients’ treatment decisions.^29,31,32^ Other factors, including lack of health care access and advanced disease, also are associated with treatment declination, consistent with our observations in this study. There are other reasons for forgoing treatment recommendations, and they may differ across racial and ethnic groups. Further research is necessary to explore and quantitatively measure these reasons and the complex interplay with socioeconomic and health care access measures that leads to racial and ethnic disparities in treatment declination among patients with BC. Closing these socioeconomic inequity gaps, patient education on treatment benefits, patient-clinician relationship building, and improved communication and share decision-making are essential to reduce the racial and ethnic disparities.

Our survival analysis results of patients who received treatment align with existing literature on racial and ethnic OS differences in the US BC population.^1,2^ OS disparities between Black and White patients remained after controlling for patient characteristics. Consistent with previous findings in patients with colorectal, breast, or ovarian cancers,^7,8,9,10,11,12^ patients with BC who forwent treatment recommendations experienced worse survival than those who received therapies. Furthermore, we found racial and ethnic disparities in OS stratified by treatment decision. In particular, among patients who declined chemotherapy, Black patients had a 7% greater mortality risk than White patients, but both groups had a similar OS if they declined radiotherapy or HT. Mortality risks were lower among American Indian, Alaska Native, or other patients, Asian or Pacific Islander patients, and Hispanic patients across all treatment cohorts. Interestingly, Black patients who declined surgery had better survival than White patients. Patients lacking access to care, with late-stage presentation or higher tumor grade, or with multiple comorbid conditions also experienced poor OS, irrespective of treatment modality. These findings suggest that treatment decisions, socioeconomic indicators, and clinical factors do not address racial and ethnic survival differences in patients with BC. Lifestyle behaviors, genetic predisposition, the environment, and other risk factors that are not collected by the NCDB could have contributed to these survival disparities, which warrants future research on the intersections of these factors and treatment declination.

### Limitations

To our knowledge, this study is the largest to date evaluating the pattern and long-term trends of and racial and ethnic disparities in treatment declination and mortality risk among patients with BC at the national level, but it has some limitations. First, underreporting is likely, given the nature of the NCDB registry, and patient perceptions toward treatment recommendations are not ascertained. Further research is necessary to explore and accurately capture the reasons why patients with BC decline recommendations. Another limitation pertains to the lack of information on whether patients sought a second opinion from other clinicians or whether patients who declined treatment later decided to receive the treatment. Third, there are unmeasured potential confounders, eg, marital status, social support, cultural backgrounds, and religious beliefs, that may play an important role in treatment decisions, affecting the racial and ethnic disparities observed, as well as patient frailty in the survival analysis. This study was also limited by not assessing declination of various specific systemic therapy regimens, as the rate probably differs; nor did were assess how these might impact other health outcomes, which is worth exploring in future studies. Additionally, the patient cohorts may not be representative of all patients with BC in the US. However, our findings were consistent with SEER population-based study results.

## Conclusions

In this nationwide cross-sectional study of patients with BC, the treatment declination rate was highest for chemotherapy and lowest for surgery, with significantly increased trends over time in HT, radiotherapy, and surgery cohorts. Patients from racial and ethnic minority groups were more likely to decline chemotherapy, radiotherapy, or surgery but less likely to decline HT than White patients. Older age, socioeconomic disparities, and advanced disease also were associated with patients’ decision to forgo treatment recommendations. Black patients who declined chemotherapy had a higher risk of mortality than White patients, while no OS difference between Black and White patients who declined HT or radiotherapy. Regardless of treatment modality, American Indian, Alaska Native, or other, Asian or Pacific Islander, and Hispanic patients had better survival. Our findings highlight racial and ethnic disparities in declination of treatment recommendations and OS, suggesting the need for equity-focused interventions, eg, patient education on treatment benefits, patient-clinician relationship building, and improved patient-clinician communication and shared decision-making, to reduce the disparities and improve patients’ survival outcomes.

## Notes

Supplement 1. **eTable 1.** Percentage of Treatment “Declined/Received” Missingness Across Patient Cohorts Over Time **eTable 2.** Distributions of Patient Characteristics by Chemotherapy Decision Comparing “Declined/Received” Not Missing vs Missing **eTable 3.** Distributions of Patient Characteristics by Hormone Therapy Decision Comparing “Declined/Received” Not Missing vs Missing **eTable 4.** Distributions of Patient Characteristics by Radiotherapy Decision Comparing “Declined/Received” Not Missing vs. Missing **eTable 5.** Distributions of Patient Characteristics by Surgery Decision Comparing “Declined/Received” Not Missing vs Missing **eTable 6.** Overall Characteristics of Patients With Breast Cancer in the National Cancer Database **eTable 7.** Sociodemographic and Clinicopathologic Factors Associated With Treatment Declination: Multivariable Logistic Regression after Inverse Probability Weighting Adjusting for Missingness of Treatment Decision **eTable 8.** Associated Characteristics With Decision on Chemotherapy in Patients With Stage I-IV Breast Cancer **eTable 9.** Distributions of Chemotherapy “Declined/Received” Among Patients With Early-Stage, HR+/*ERBB2*- Breast Cancer Post-Surgery, by Multigene Assay Testing Result **eTable 10.** Associated Characteristics With Decision on Hormone Therapy in Patients With Stage I-IV, Hormone Receptor–Positive Breast Cancer **eTable 11.** Associated Characteristics With Decision on Radiation Therapy in Patients With Stage I-III Breast Cancer **eTable 12.** Associated Characteristics With Decision on Surgery in Patients With Stage I-III Breast Cancer **eTable 13.** Kaplan-Meier Estimates of Median Overall Survival Time in Breast Cancer Patients Stratified By Treatment Decision and Race and Ethnicity **eTable 14.** Kaplan-Meier Estimated 5-Year and 10-Year Overall Survival of Breast Cancer Patients Stratified by Treatment Decision **eTable 15.** Associated Factors With Overall Survival in Patients With Stage I-IV Breast Cancer by Treatment Decision on Chemotherapy **eTable 16.** Associated Factors With Overall Survival in Patients With Stage I-IV, HR-Positive Breast Cancer by Treatment Decision on Hormone Therapy **eTable 17.** Associated Factors With Overall Survival in Patients With Stage I-III Breast Cancer by Treatment Decision on Radiotherapy **eTable 18.** Associated Factors With Overall Survival in Patients With Stage I-III Breast Cancer by Treatment Decision on Surgery **eFigure.** Kaplan-Meier Curves for Overall Survival Stratified by Race and Ethnicity in Patients Who Received Treatment

Supplement 2.
Data Sharing Statement
