# Supplementary material for: Declination of Treatment, Racial and Ethnic Disparity, and Overall Survival in US Patients With Breast Cancer
Source: JAMA Netw Open. 2024 May 9;7(5):e249449. doi: 10.1001/jamanetworkopen.2024.9449 (PMC11082691; doi:10.1001/jamanetworkopen.2024.9449)
Supplement: Supplement 1. — eTable 1. Percentage of Treatment “Declined/Received” Missingness Across Patient Cohorts Over Time eTable 2. Distributions of Patient Characteristics by Chemotherapy Decision Comparing “Declined/Received” Not Missing vs Missing eTable 3. Distributions of Patient Characteristics by Hormone Therapy Decision Comparing “Declined/Received” Not Missing vs Missing eTable 4. Distributions of Patient Characteristics by Radiotherapy Decision Comparing “Declined/Received” Not Missing vs. Missing eTable 5. Distributions of Patient Characteristics by Surgery Decision Comparing “Declined/Received” Not Missing vs Missing eTable 6. Overall Characteristics of Patients With Breast Cancer in the National Cancer Database eTable 7. Sociodemographic and Clinicopathologic Factors Associated With Treatment Declination: Multivariable Logistic Regression after Inverse Probability Weighting Adjusting for Missingness of Treatment Decision eTable 8. Associated Characteristics With Decision on Chemotherapy in Patients With Stage I-IV Breast Cancer eTable 9. Distributions of Chemotherapy “Declined/Received” Among Patients With Early-Stage, HR+/ERBB2- Breast Cancer Post-Surgery, by Multigene Assay Testing Result eTable 10. Associated Characteristics With Decision on Hormone Therapy in Patients With Stage I-IV, Hormone Receptor–Positive Breast Cancer eTable 11. Associated Characteristics With Decision on Radiation Therapy in Patients With Stage I-III Breast Cancer eTable 12. Associated Characteristics With Decision on Surgery in Patients With Stage I-III Breast Cancer eTable 13. Kaplan-Meier Estimates of Median Overall Survival Time in Breast Cancer Patients Stratified By Treatment Decision and Race and Ethnicity eTable 14. Kaplan-Meier Estimated 5-Year and 10-Year Overall Survival of Breast Cancer Patients Stratified by Treatment Decision eTable 15. Associated Factors With Overall Survival in Patients With Stage I-IV Breast Cancer by Treatment Decision on Chemotherapy eTable 16. Associated Factors [file jamanetwopen-e249449-s001.pdf]

Supplemental Online Content

Freeman JQ, Li JL, Fisher SG, Yao KA, David SP, Huo D. Declination of treatment, racial and ethnic disparity, and overall survival in US patients with breast cancer. *JAMA Netw Open*. 2024;7(5):e249449. doi:10.1001/jamanetworkopen.2024.9449

eTable 1. Percentage of Treatment “Declined/Received” Missingness Across Patient Cohorts Over Time
eTable 2. Distributions of Patient Characteristics by Chemotherapy Decision Comparing “Declined/Received” Not Missing vs Missing
eTable 3. Distributions of Patient Characteristics by Hormone Therapy Decision Comparing “Declined/Received” Not Missing vs Missing
eTable 4. Distributions of Patient Characteristics by Radiotherapy Decision Comparing “Declined/Received” Not Missing vs Missing
eTable 5. Distributions of Patient Characteristics by Surgery Decision Comparing “Declined/Received” Not Missing vs Missing
eTable 6. Overall Characteristics of Patients With Breast Cancer in the National Cancer Database
eTable 7. Sociodemographic and Clinicopathologic Factors Associated With Treatment Declination: Multivariable Logistic Regression After Inverse Probability Weighting Adjusting for Missingness of Treatment Decision
eTable 8. Associated Characteristics With Decision on Chemotherapy in Patients With Stage I-IV Breast Cancer
eTable 9. Distributions of Chemotherapy “Declined/Received” Among Patients With Early-Stage, HR+/*ERBB2*- Breast Cancer Post-Surgery, by Multigene Assay Testing Result
eTable 10. Associated Characteristics With Decision on Hormone Therapy in Patients With Stage I-IV, Hormone Receptor–Positive Breast Cancer
eTable 11. Associated Characteristics With Decision on Radiation Therapy in Patients With Stage I-III Breast Cancer
eTable 12. Associated Characteristics With Decision on Surgery in Patients With Stage I-III Breast Cancer
eTable 13. Kaplan-Meier Estimates of Median Overall Survival Time in Breast Cancer Patients Stratified By Treatment Decision and Race/Ethnicity
eTable 14. Kaplan-Meier Estimated 5-Year and 10-Year Overall Survival of Breast Cancer Patients Stratified by Treatment Decision
eTable 15. Associated Factors With Overall Survival in Patients With Stage I-IV Breast Cancer by Treatment Decision on Chemotherapy
eTable 16. Associated Factors With Overall Survival in Patients With Stage I-IV, HR-Positive Breast Cancer by Treatment Decision on Hormone Therapy
eTable 17. Associated Factors With Overall Survival in Patients With Stage I-III Breast Cancer by Treatment Decision on Radiotherapy
eTable 18. Associated Factors With Overall Survival in Patients With Stage I-III Breast Cancer by Treatment Decision on Surgery
eFigure. Kaplan-Meier Curves for Overall Survival Stratified by Race and Ethnicity in Patients Who Received Treatment

This supplemental material has been provided by the authors to give readers additional information about their work.

© 2024 Freeman JQ et al. *JAMA Network Open.*

eTable 1. Percentage of Treatment “Declined/Received” Missingness Across Patient Cohorts Over Time

	**Chemotherapy cohort**	**Hormone therapy cohort**	**Radiotherapy cohort**	**Surgery cohort**
**Year of diagnosis**	**Missing, n (%)**	**Missing, n (%)**	**Missing, n (%)**	**Missing, n (%)**
2004	2,382 (3.9)	2,920 (4.7)	2,366 (3.4)	330 (0.3)
2005	2,382 (3.7)	3,139 (4.7)	2,205 (3.1)	348 (0.3)
2006	2,148 (3.3)	2,964 (4.0)	1,845 (2.5)	318 (0.3)
2007	2,005 (2.9)	3,008 (3.7)	1,824 (2.3)	304 (0.2)
2008	2,096 (2.9)	3,234 (3.7)	1,986 (2.4)	461 (0.4)
2009	1,939 (2.6)	3,023 (3.1)	1,819 (2.1)	525 (0.4)
2010	2,069 (2.7)	2,947 (2.8)	1,670 (1.9)	582 (0.4)
2011	2,029 (2.6)	3,025 (2.7)	1,731 (1.9)	682 (0.5)
2012	1,584 (2.0)	3,091 (2.6)	1,773 (1.8)	722 (0.5)
2013	1,501 (1.8)	3,221 (2.5)	1,782 (1.7)	898 (0.6)
2014	1,536 (1.8)	3,742 (2.8)	1,657 (1.5)	1,032 (0.6)
2015	1,491 (1.7)	3,976 (2.9)	1,575 (1.4)	1,142 (0.7)
2016	1,413 (1.6)	4,110 (2.8)	1,658 (1.4)	1,331 (0.8)
2017	1,560 (1.7)	4,632 (3.1)	1,653 (1.4)	1,381 (0.8)
2018	1,187 (1.4)	3,419 (2.2)	1,063 (0.9)	975 (0.5)
2019	1,088 (1.3)	4,406 (2.8)	1,269 (1.0)	1,257 (0.7)
2020	1,195 (1.6)	4,637 (3.4)	1,570 (1.5)	1,530 (0.9)

© 2024 Freeman JQ et al. *JAMA Network Open.*

eTable 2. Distributions of Patient Characteristics by Chemotherapy Decision Comparing “Declined/Received” Not missing vs. Missing

	Chemotherapy “declined/received”		
	**NOT missing, n (%)**	**Missing, n (%)**	***P* value [a]**
**Total**	1296488 (97.8)	29521 (2.2)	
**Age at diagnosis,** mean (SD)	56.5 (12.5)	62.4 (13.6)	<0.001
**Age at diagnosis,** median (IQR)	56.0 (47.0, 65.0)	62.0 (52.0, 73.0)	<0.001
**Sex**			<0.001
Male	11455 (0.9)	432 (1.5)	
Female	1285033 (99.1)	29089 (98.5)	
**Race/ethnicity**			<0.001
White	955135 (73.7)	20478 (69.4)	
Black	182706 (14.1)	4390 (14.9)	
Asian or Pacific Islander	50771 (3.9)	1165 (3.9)	
Hispanic	85661 (6.6)	2518 (8.5)	
American India, Alaska Native, or Other	22215 (1.7)	970 (3.3)	
**Type of health insurance**			<0.001
Uninsured	33986 (2.6)	1023 (3.5)	
Private or managed care	770370 (59.4)	13505 (45.7)	
Medicaid	112960 (8.7)	2022 (6.8)	
Medicare	341275 (26.3)	11578 (39.2)	
Other government or unknown	37897 (2.9)	1393 (4.7)	
**Median household income quartiles [b]**			<0.001
<$40,227	192080 (16.8)	4867 (18.2)	
$40,227–$50,353	233812 (20.4)	4957 (18.5)	
$50,354–$63,332	264318 (23.1)	5584 (20.9)	
≥$63,333	453296 (39.6)	11339 (42.4)	
**Rural/urban area [c]**			<0.001
Metro	1094444 (86.8)	25882 (91.1)	
Urban	147471 (11.7)	2257 (7.9)	
Rural	19247 (1.5)	281 (1.0)	
**Facility type**			<0.001

© 2024 Freeman JQ et al. *JAMA Network Open.*

Community	86162 (7.3)	2120 (7.5)	
Comprehensive community	485796 (41.1)	10357 (36.7)	
Academic or research	361638 (30.6)	9585 (34.0)	
Integrated network	248832 (21.0)	6170 (21.9)	
**Charlson-Deyo comorbidity index**			<0.001
0	1104887 (85.2)	25080 (85.0)	
1	149860 (11.6)	3373 (11.4)	
≥2	41741 (3.2)	1068 (3.6)	
**Histologic type**			<0.001
Ductal	1044349 (80.6)	22621 (76.6)	
Lobular	102906 (7.9)	2864 (9.7)	
Ductal and lobular	58628 (4.5)	1474 (5.0)	
Other	90605 (7.0)	2562 (8.7)	
**AJCC stage group**			<0.001
1	416538 (32.1)	14224 (48.2)	
2	547928 (42.3)	9872 (33.4)	
3	241718 (18.6)	3044 (10.3)	
4	90304 (7.0)	2381 (8.1)	
**Molecular subtype**			<0.001
HR+/ERBB2-	454888 (52.5)	9923 (66.6)	
HR+/ERBB2+	155969 (18.0)	1781 (12.0)	
HR-/ERBB2+	69111 (8.0)	884 (5.9)	
TNBC	186855 (21.6)	2308 (15.5)	
**Tumor grade**			<0.001
1	113085 (9.4)	5118 (19.4)	
2	479199 (39.7)	11797 (44.7)	
3	613850 (50.9)	9482 (35.9)	

Abbreviations: SD, standard deviation; IQR; interquartile range; AJCC, American Joint Committee on Cancer; HR, hormone receptor; ERBB2, human epidermal growth factor receptor 2; TNBC, triple-negative breast cancer.

[a] *P* values were computed using Student's t, Wilcoxon rank-sum, or Pearson's Chi-square tests.

[b] Based on the 2016 American Community Survey data, spanning years 2012–2016 and adjusted for 2016 inflation.

[c] Measured by matching the state and county FIPS code of the patient recorded at the time of diagnosis against 2013 files published by the United States Department of Agriculture Economic Research Service.

© 2024 Freeman JQ et al. *JAMA Network Open.*

eTable 3. Distributions of Patient Characteristics by Hormone Therapy Decision Comparing "Declined/Received" Not missing vs. Missing

	**Hormone therapy "declined/received"**		
	**NOT missing, n (%)**	**Missing, n (%)**	***P* value [a]**
**Total**	1893339 (96.9)	59494 (3.1)	
**Age at diagnosis,** mean (SD)	61.8 (13.0)	61.4 (13.4)	<0.001
**Age at diagnosis,** median (IQR)	62.0 (52.0, 71.0)	62.0 (51.0, 71.0)	<0.001
**Sex**			<0.001
Male	16914 (0.9)	932 (1.6)	
Female	1876425 (99.1)	58562 (98.4)	
**Race/ethnicity**			<0.001
White	1524505 (80.5)	44214 (74.3)	
Black	174272 (9.2)	6995 (11.8)	
Asian or Pacific Islander	67835 (3.6)	2471 (4.2)	
Hispanic	97176 (5.1)	4183 (7.0)	
American India, Alaska Native, or Other	29551 (1.6)	1631 (2.7)	
**Type of health insurance**			<0.001
Uninsured	30326 (1.6)	1404 (2.4)	
Private or managed care	953742 (50.4)	29091 (48.9)	
Medicaid	108128 (5.7)	4132 (6.9)	
Medicare	757349 (40.0)	22817 (38.4)	
Other government or unknown	43794 (2.3)	2050 (3.4)	
**Median household income quartiles [b]**			<0.001
<$40,227	239816 (14.5)	7866 (14.9)	
$40,227–$50,353	329905 (19.9)	9336 (17.7)	
$50,354–$63,332	388322 (23.4)	11210 (21.2)	
≥$63,333	698493 (42.2)	24417 (46.2)	
**Rural/urban area [c]**			<0.001
Metro	1599687 (86.7)	51824 (90.3)	
Urban	217134 (11.8)	4982 (8.7)	
Rural	28610 (1.6)	560 (1.0)	
**Facility type**			<0.001

© 2024 Freeman JQ et al. *JAMA Network Open.*

Community	128834 (7.1)	4214 (7.4)	
Comprehensive community	760598 (41.9)	21610 (38.2)	
Academic or research	535287 (29.5)	17512 (30.9)	
Integrated network	391192 (21.5)	13278 (23.5)	
**Charlson-Deyo comorbidity index**			<0.001
0	1574130 (83.1)	50868 (85.5)	
1	240913 (12.7)	6617 (11.1)	
≥2	78296 (4.1)	2009 (3.4)	
**Histologic type**			<0.001
Ductal	1443493 (76.2)	45976 (77.3)	
Lobular	231266 (12.2)	6233 (10.5)	
Ductal and lobular	114214 (6.0)	3410 (5.7)	
Other	104366 (5.5)	3875 (6.5)	
**AJCC stage group**			<0.001
1	1135275 (60.0)	36126 (60.7)	
2	520891 (27.5)	15656 (26.3)	
3	163500 (8.6)	5665 (9.5)	
4	73673 (3.9)	2047 (3.4)	
**ERBB2 status**			<0.001
Negative	1250585 (88.8)	33172 (84.2)	
Positive	157103 (11.2)	6241 (15.8)	
**Tumor grade**			<0.001
1	495152 (28.0)	14279 (26.2)	
2	904782 (51.2)	27096 (49.7)	
3	367167 (20.8)	13194 (24.2)	

Abbreviations: SD, standard deviation; IQR; interquartile range; AJCC, American Joint Committee on Cancer; ERBB2, human epidermal growth factor receptor 2.
[a] *P* values were computed using Student's t, Wilcoxon rank-sum, or Pearson's Chi-square tests.
[b] Based on the 2016 American Community Survey data, spanning years 2012–2016 and adjusted for 2016 inflation.
[c] Measured by matching the state and county FIPS code of the patient recorded at the time of diagnosis against 2013 files published by the United States Department of Agriculture Economic Research Service.

© 2024 Freeman JQ et al. *JAMA Network Open.*

eTable 4. Distributions of Patient Characteristics by Radiotherapy Decision Comparing “Declined/Received” Not missing vs. Missing

	**Radiotherapy “declined/received”**		
	**NOT missing, n (%)**	**Missing, n (%)**	***P* value [a]**
**Total**	1635916 (98.2)	29446 (1.8)	
**Age at diagnosis,** mean (SD)	60.7 (12.4)	60.9 (14.1)	0.004
**Age at diagnosis,** median (IQR)	61.0 (52.0, 69.0)	61.0 (50.0, 72.0)	0.016
**Sex**			<0.001
Male	9241 (0.6)	354 (1.2)	
Female	1626675 (99.4)	29092 (98.8)	
**Race/ethnicity**			<0.001
White	1288646 (78.8)	20585 (69.9)	
Black	177711 (10.9)	4238 (14.4)	
Asian or Pacific Islander	56601 (3.5)	1206 (4.1)	
Hispanic	86865 (5.3)	2552 (8.7)	
American India, Alaska Native, or Other	26093 (1.6)	865 (2.9)	
**Type of health insurance**			<0.001
Uninsured	27882 (1.7)	998 (3.4)	
Private or managed care	870694 (53.2)	13713 (46.6)	
Medicaid	100775 (6.2)	2489 (8.5)	
Medicare	597456 (36.5)	10834 (36.8)	
Other government or unknown	39109 (2.4)	1412 (4.8)	
**Median household income quartiles [b]**			<0.001
<$40,227	211452 (14.7)	4698 (17.9)	
$40,227–$50,353	285005 (19.8)	5002 (19.1)	
$50,354–$63,332	335904 (23.4)	5667 (21.6)	
≥$63,333	603645 (42.0)	10825 (41.3)	
**Rural/urban area [c]**			<0.001
Metro	1390915 (87.2)	25308 (89.6)	
Urban	182008 (11.4)	2658 (9.4)	
Rural	23027 (1.4)	288 (1.0)	
**Facility type**			<0.001
Community	114587 (7.3)	2034 (7.4)	

© 2024 Freeman JQ et al. *JAMA Network Open.*

Comprehensive community	659995 (42.2)	9331 (33.9)	
Academic or research	451544 (28.9)	10421 (37.8)	
Integrated network	337154 (21.6)	5769 (20.9)	
**Charlson-Deyo comorbidity index**			0.059
0	1384008 (84.6)	25016 (85.0)	
1	194716 (11.9)	3376 (11.5)	
≥2	57192 (3.5)	1054 (3.6)	
**Histologic type**			<0.001
Ductal	1296492 (79.3)	22796 (77.4)	
Lobular	157922 (9.7)	2763 (9.4)	
Ductal and lobular	82407 (5.0)	1521 (5.2)	
Other	99095 (6.1)	2366 (8.0)	
**AJCC stage group**			<0.001
1	957249 (58.5)	13135 (44.6)	
2	472577 (28.9)	11075 (37.6)	
3	206090 (12.6)	5236 (17.8)	
**Molecular subtype**			<0.001
HR+/ERBB2-	863080 (75.7)	10763 (67.1)	
HR+/ERBB2+	108958 (9.6)	1835 (11.4)	
HR-/ERBB2+	41298 (3.6)	927 (5.8)	
TNBC	126871 (11.1)	2517 (15.7)	
**Tumor grade**			<0.001
1	379907 (24.8)	5021 (18.6)	
2	691374 (45.1)	11571 (42.8)	
3	462990 (30.2)	10427 (38.6)	

Abbreviations: SD, standard deviation; IQR; interquartile range; AJCC, American Joint Committee on Cancer; HR, hormone receptor; ERBB2, human epidermal growth factor receptor 2; TNBC, triple-negative breast cancer.

[a] *P* values were computed using Student's t, Wilcoxon rank-sum, or Pearson's Chi-square tests.

[b] Based on the 2016 American Community Survey data, spanning years 2012–2016 and adjusted for 2016 inflation.

[c] Measured by matching the state and county FIPS code of the patient recorded at the time of diagnosis against 2013 files published by the United States Department of Agriculture Economic Research Service.

© 2024 Freeman JQ et al. *JAMA Network Open.*

eTable 5. Distributions of Patient Characteristics by Surgery Decision Comparing "Declined/Received" Not missing vs. Missing

	**Surgery "declined/received"**		
	**NOT missing, n (%)**	**Missing, n (%)**	***P* value [a]**
**Total**	2590963 (99.5)	13818 (0.5)	
**Age at diagnosis,** mean (SD)	61.3 (13.2)	59.8 (14.6)	<0.001
**Age at diagnosis,** median (IQR)	62.0 (52.0, 71.0)	59.0 (49.0, 70.0)	<0.001
**Sex**			0.67
Male	23000 (0.9)	118 (0.9)	
Female	2567963 (99.1)	13700 (99.1)	
**Race/ethnicity**			<0.001
White	2039285 (78.7)	8556 (61.9)	
Black	275297 (10.6)	2680 (19.4)	
Asian or Pacific Islander	92069 (3.6)	728 (5.3)	
Hispanic	142294 (5.5)	1344 (9.7)	
American India, Alaska Native, or Other	42018 (1.6)	510 (3.7)	
**Type of health insurance**			<0.001
Uninsured	43017 (1.7)	772 (5.6)	
Private or managed care	1324312 (51.1)	6302 (45.6)	
Medicaid	152744 (5.9)	1468 (10.6)	
Medicare	1005973 (38.8)	4605 (33.3)	
Other government or unknown	64917 (2.5)	671 (4.9)	
**Median household income quartiles [b]**			<0.001
<$40,227	347712 (15.2)	2253 (18.5)	
$40,227–$50,353	457134 (20.0)	2217 (18.2)	
$50,354–$63,332	531045 (23.3)	2547 (20.9)	
≥$63,333	946768 (41.5)	5145 (42.3)	
**Rural/urban area [c]**			<0.001
Metro	2194393 (87.0)	12255 (91.5)	
Urban	290556 (11.5)	1005 (7.5)	
Rural	37793 (1.5)	132 (1.0)	
**Facility type**			<0.001
Community	181025 (7.3)	1049 (8.3)	

© 2024 Freeman JQ et al. *JAMA Network Open.*

Comprehensive community	1036243 (42.0)	4191 (33.1)	
Academic or research	716357 (29.0)	5003 (39.5)	
Integrated network	533359 (21.6)	2408 (19.0)	
**Charlson-Deyo comorbidity index**			<0.001
0	2161792 (83.4)	12414 (89.8)	
1	326811 (12.6)	973 (7.0)	
≥2	102360 (4.0)	431 (3.1)	
**Histologic type**			<0.001
Ductal	2035573 (78.6)	10931 (79.1)	
Lobular	259520 (10.0)	1209 (8.7)	
Ductal and lobular	134918 (5.2)	459 (3.3)	
Other	160952 (6.2)	1219 (8.8)	
**AJCC stage group**			<0.001
1	1538942 (59.4)	5622 (40.7)	
2	787987 (30.4)	5456 (39.5)	
3	264034 (10.2)	2740 (19.8)	
**Molecular subtype**			<0.001
HR+/ERBB2-	1317276 (74.9)	6523 (61.4)	
HR+/ERBB2+	171382 (9.7)	1439 (13.5)	
HR-/ERBB2+	69822 (4.0)	733 (6.9)	
TNBC	201250 (11.4)	1925 (18.1)	
**Tumor grade**			<0.001
1	588515 (24.3)	2079 (17.0)	
2	1093700 (45.2)	5205 (42.5)	
3	736619 (30.5)	4971 (40.6)	

Abbreviations: SD, standard deviation; IQR; interquartile range; AJCC, American Joint Committee on Cancer; HR, hormone receptor; ERBB2, human epidermal growth factor receptor 2; TNBC, triple-negative breast cancer.

[a] *P* values were computed using Student's t, Wilcoxon rank-sum, or Pearson's Chi-square tests.

[b] Based on the 2016 American Community Survey data, spanning years 2012–2016 and adjusted for 2016 inflation.

[c] Measured by matching the state and county FIPS code of the patient recorded at the time of diagnosis against 2013 files published by the United States Department of Agriculture Economic Research Service.

© 2024 Freeman JQ et al. *JAMA Network Open.*

eTable 6. Overall Characteristics of Patients With Breast Cancer in the National Cancer Database

**Variable**	**Total (N=2,837,446)**
	No. (row %)
**Age at diagnosis (years)**	
mean (SD)	61.6 (13.4)
Median (IQR)	62.0 (52.0, 71.0)
**Sex**	
Male	26231 (0.9)
Female	2811215 (99.1)
**Race/Ethnicity**	
White	2213914 (78.0)
Black	317258 (11.2)
Asian or Pacific Islander	100251 (3.5)
Hispanic	158428 (5.6)
American Indian, Alaska Native, or Other	47595 (1.7)
**Type of health insurance**	
Uninsured	53969 (1.9)
Private or managed care	1414826 (49.9)
Medicaid	177824 (6.3)
Medicare	1115442 (39.3)
Other government or unknown	75385 (2.7)
**Median household income quartiles** [a]	
<$40,227	391557 (15.6)
$40,227–$50,353	503202 (20.1)
$50,354–$63,332	580650 (23.2)
≥$63,333	1026795 (41.0)
**Rural/urban area** [b]	
Metropolitan	2405390 (87.1)
Urban	316053 (11.4)
Rural	41191 (1.5)
**Type of cancer program**	
Community	200322 (7.4)
Comprehensive community	1126191 (41.7)
Academic or research	793875 (29.4)

© 2024 Freeman JQ et al. *JAMA Network Open.*

Integrated network	578752 (21.4)
**Charlson-Deyo comorbidity score**	
0	2363067 (83.3)
1	356281 (12.6)
≥2	118098 (4.2)
**Histologic type**	
Ductal	2200119 (77.5)
Lobular	284305 (10.0)
Ductal and lobular	143118 (5.0)
Other	209904 (7.4)
**AJCC stage group**	
I	1578116 (55.6)
II	823827 (29.0)
III	286572 (10.1)
IV	148931 (5.2)
**Molecular subtype**	
HR+/ERBB2-	1424349 (74.0)
HR+/ERBB2+	194496 (10.1)
HR-/ERBB2+	81870 (4.3)
TNBC	225138 (11.7)
**Tumor grade**	
1	612864 (23.5)
2	1177279 (45.1)
3	820978 (31.4)

Abbreviations: No., number; SD, standard deviation; IQR; interquartile range; AJCC, American Joint Committee on Cancer; HR, hormone receptor; ERBB2, human epidermal growth factor receptor 2; TNBC, triple-negative breast cancer.

[a] Based on the 2016 American Community Survey data, spanning years 2012–2016 and adjusted for 2016 inflation.

[b] Measured by matching the state and county FIPS code of the patient recorded at the time of diagnosis against 2013 files published by the United States Department of Agriculture Economic Research Service.

© 2024 Freeman JQ et al. *JAMA Network Open.*

eTable 7. Sociodemographic and Clinicopathologic Factors Associated with Treatment Declination: Multivariable Logistic Regression after Inverse Probability Weighting adjusting for missingness of treatment decision

	**Chemotherapy cohort** [a]	**Hormone therapy cohort** [b]	**Radiotherapy cohort** [c]	**Surgery cohort** [c]
**Variable**	**AOR (95% CI)** [d]	**AOR (95% CI)** [e]	**AOR (95% CI)** [d]	**AOR (95% CI)** [d]
**Race/Ethnicity**				
American Indian, Alaska Native, or Other [f]	1.13 (1.05-1.21)	1.04 (0.96-1.11)	1.02 (0.94-1.09)	1.47 (1.26-1.72)
Asian or Pacific Islander	1.21 (1.16-1.27)	0.81 (0.77-0.85)	1.01 (0.96-1.06)	1.29 (1.15-1.44)
Black	1.03 (1.00-1.06)	0.86 (0.83-0.89)	1.05 (1.02-1.08)	2.01 (1.89-2.14)
Hispanic	0.78 (0.75-0.82)	0.66 (0.63-0.69)	0.74 (0.70-0.77)	0.79 (0.71-0.89)
White	1.0 (reference)	1.0 (reference)	1.0 (reference)	1.0 (reference)
**Age at diagnosis** (per 10-year increase)	2.39 (2.36-2.42)	1.44 (1.42-1.45)	2.08 (2.05-2.10)	2.83 (2.75-2.91)
**Sex**				
Male	1.0 (reference)	1.0 (reference)	1.0 (reference)	1.0 (reference)
Female	1.35 (1.23-1.47)	1.35 (1.22-1.49)	1.04 (0.94-1.16)	2.02 (1.59-2.57)
**Type of health insurance**				
Uninsured	1.60 (1.50-1.72)	1.60 (1.49-1.73)	1.96 (1.82-2.12)	4.81 (4.20-5.51)
Private or managed care	1.0 (reference)	1.0 (reference)	1.0 (reference)	1.0 (reference)
Medicaid	1.51 (1.45-1.57)	1.43 (1.38-1.50)	1.86 (1.79-1.94)	3.18 (2.90-3.49)
Medicare	1.03 (1.01-1.06)	1.02 (0.99-1.04)	1.09 (1.07-1.12)	0.94 (0.89-1.00)
Other government or unknown	1.02 (0.96-1.09)	1.00 (0.93-1.07)	1.09 (1.02-1.16)	1.17 (1.00-1.38)
**Median household income quartiles** [g]				
<$40,227	1.04 (1.01-1.07)	0.87 (0.84-0.90)	1.16 (1.13-1.20)	1.14 (1.07-1.22)
$40,227–$50,353	1.06 (1.03-1.09)	0.95 (0.92-0.97)	1.11 (1.08-1.13)	1.13 (1.07-1.20)
$50,354–$63,332	1.05 (1.03-1.08)	1.00 (0.98-1.02)	1.09 (1.07-1.12)	1.06 (1.00-1.12)
≥$63,333	1.0 (reference)	1.0 (reference)	1.0 (reference)	1.0 (reference)
**Rural/urban area** [h]				
Metropolitan	1.0 (reference)	1.0 (reference)	1.0 (reference)	1.0 (reference)
Urban	0.94 (0.92-0.97)	0.96 (0.93-0.99)	1.01 (0.99-1.04)	0.83 (0.77-0.89)
Rural	0.92 (0.85-0.98)	0.96 (0.90-1.03)	1.05 (0.98-1.12)	0.68 (0.55-0.83)
**Type of cancer program**				
Community	0.94 (0.91-0.98)	1.07 (1.03-1.11)	0.92 (0.89-0.95)	0.89 (0.82-0.97)

© 2024 Freeman JQ et al. *JAMA Network Open.*

Comprehensive community	1.10 (1.08-1.13)	1.19 (1.16-1.22)	1.00 (0.98-1.02)	0.88 (0.83-0.92)
Academic or research	1.0 (reference)	1.0 (reference)	1.0 (reference)	1.0 (reference)
Integrated network	1.03 (1.00-1.05)	1.13 (1.10-1.16)	1.03 (1.01-1.06)	0.90 (0.85-0.96)
**Charlson-Deyo comorbidity index**				
0	1.0 (reference)	1.0 (reference)	1.0 (reference)	1.0 (reference)
1	0.98 (0.95-1.00)	0.89 (0.87-0.91)	1.06 (1.03-1.08)	0.75 (0.70-0.80)
≥2	1.24 (1.20-1.29)	1.03 (0.99-1.07)	1.38 (1.34-1.43)	1.03 (0.96-1.11)
**AJCC stage group**				
I	1.0 (reference)	1.0 (reference)	1.0 (reference)	1.0 (reference)
II	0.49 (0.48-0.50)	0.73 (0.71-0.75)	1.26 (1.23-1.28)	2.69 (2.56-2.83)
III	0.26 (0.25-0.26)	0.59 (0.56-0.61)	1.20 (1.16-1.23)	4.32 (4.06-4.61)
IV	0.25 (0.24-0.26)	0.31 (0.29-0.34)	——	——
**Tumor grade**				
1	2.30 (2.24-2.37)	1.19 (1.16-1.22)	1.21 (1.17-1.24)	1.33 (1.24-1.43)
2	1.51 (1.48-1.54)	0.93 (0.91-0.96)	1.04 (1.01-1.06)	1.27 (1.20-1.34)
3	1.0 (reference)	1.0 (reference)	1.0 (reference)	1.0 (reference)

Abbreviations: AOR, adjusted odds ratio; CI, confidence interval; AJCC, American Joint Committee on Cancer; ERBB2, human epidermal growth factor receptor 2.

[a] among patients with stage I-IV breast cancer.

[b] among patients with stage I-IV, hormone receptor-positive breast cancer.

[c] among patients with stage I-III breast cancer.

[d] Additionally adjusted for histologic type, molecular subtype, and year of initial diagnosis.

[e] Additionally adjusted for histologic type, ERBB2 status, and year of initial diagnosis.

[f] Other is a racial/ethnic group listed in the National Cancer Database (NCDB) and represents patients who were classified as Other by local cancer registries. The NCDB does not specifically define race/ethnicity classified into Other.

[g] Based on the 2016 American Community Survey data, spanning years 2012–2016 and adjusted for 2016 inflation.

[h] Measured by matching the state and county FIPS code of the patient recorded at the time of diagnosis against 2013 files published by the United States Department of Agriculture Economic Research Service.

© 2024 Freeman JQ et al. *JAMA Network Open.*

eTable 8. Associated Characteristics with Decision on Chemotherapy in Patients With Stage I-IV Breast Cancer

	**Decision on chemotherapy (N=1,296,488)**				**Reduced Model**	**Full Model**
**Variable**	**Received** (n=1,171,767 [90.4%]) No. (row %)	**Declined** (n=124,721 [9.6%]) No. (row %)	***P* value** [a]	**Crude OR (95% CI)**	**AOR (95% CI)**	**AOR (95% CI)** [b]
**Age at diagnosis (years)**, mean (SD)	55.4 (11.9)	66.5 (13.1)	<.001			
Per 10-year increase				2.16 (2.15-2.17)	2.21 (2.19-2.23)	2.38 (2.35-2.40)
**Sex**						
Male	10,292 (89.9)	1,163 (10.2)	.052	1.0 (reference)	——	1.0 (reference)
Female	1,161,475 (90.4)	123,558 (9.6)		0.94 (0.89-1.001)	——	1.34 (1.23-1.47)
**Race/Ethnicity**						
White	856,473 (89.7)	98,662 (10.3)	<.001	1.0 (reference)	1.0 (reference)	1.0 (reference)
Black	167,952 (91.9)	14,754 (8.1)		0.76 (0.75-0.78)	1.09 (1.06-1.11)	1.03 (1.01-1.06)
Asian or Pacific Islander	46,311 (91.2)	4,460 (8.8)		0.84 (0.81-0.86)	1.24 (1.19-1.29)	1.21 (1.16-1.27)
Hispanic	80,745 (94.3)	4,916 (5.7)		0.53 (0.51-0.54)	0.83 (0.80-0.87)	0.78 (0.75-0.82)
American Indian, Alaska Native, or Other	20,286 (91.3)	1,929 (8.7)		0.83 (0.79-0.87)	1.14 (1.07-1.21)	1.13 (1.05-1.21)
**Type of health insurance**						
Uninsured	31,555 (92.9)	2,431 (7.2)	<.001	1.21 (1.16-1.26)	——	1.61 (1.51-1.72)
Private or managed care	724,102 (94.0)	46,268 (6.0)		1.0 (reference)	——	1.0 (reference)
Medicaid	105,342 (93.3)	7,618 (6.7)		1.13 (1.10-1.16)	——	1.51 (1.46-1.57)
Medicare	275,447 (80.7)	65,828 (19.3)		3.74 (3.69-3.79)	——	1.04 (1.01-1.06)
Other government or unknown	35,321 (93.2)	2,576 (6.8)		1.14 (1.10-1.19)	——	1.02 (0.96-1.09)
**Median household income quartiles** [c]						
<$40,227	173,871 (90.5)	18,209 (9.5)	<.001	1.04 (1.02-1.06)	——	1.04 (1.01-1.07)
$40,227–$50,353	210,350 (90.0)	23,462 (10.0)		1.11 (1.09-1.13)	——	1.06 (1.03-1.09)
$50,354–$63,332	238,048 (90.1)	26,270 (9.9)		1.10 (1.08-1.11)	——	1.05 (1.03-1.08)
≥$63,333	411,837 (90.9)	41,459 (9.2)		1.0 (reference)	——	1.0 (reference)
**Rural/urban area** [d]						
Metropolitan	989,347 (90.4)	105,097 (9.6)	<.001	1.0 (reference)	——	1.0 (reference)
Urban	132,679 (90.0)	14,792 (10.0)		1.05 (1.03-1.07)	——	0.94 (0.92-0.97)
Rural	17,282 (89.8)	1,965 (10.2)		1.07 (1.02-1.12)	——	0.92 (0.85-0.98)
**Type of cancer program**						

© 2024 Freeman JQ et al. *JAMA Network Open.*

Community	76,534 (88.8)	9,628 (11.2)	<.001	1.29 (1.26-1.33)	——	0.94 (0.91-0.98)
Comprehensive community	431,000 (88.7)	54,796 (11.3)		1.31 (1.29-1.33)	——	1.10 (1.08-1.13)
Academic or research	329,595 (91.1)	32,043 (8.9)		1.0 (reference)	——	1.0 (reference)
Integrated network	223,289 (89.7)	25,543 (10.3)		1.18 (1.16-1.20)	——	1.03 (1.00-1.05)
**Charlson-Deyo comorbidity score**						
0	1,005,164 (91.0))	99,723 (9.0)	<.001	1.0 (reference)	1.0 (reference)	1.0 (reference)
1	132,069 (88.1)	17,791 (11.9)		1.36 (1.33-1.38)	0.98 (0.96-1.00)	0.98 (0.95-1.00)
≥2	34,534 (82.7)	7,027 (17.3)		2.10 (2.05-2.16)	1.22 (1.17-1.26)	1.24 (1.20-1.29)
**Histologic type**						
Ductal	948,541 (90.8)	95,808 (9.2)	<.001	1.0 (reference)	1.0 (reference)	1.0 (reference)
Lobular	89,948 (87.4)	12,958 (12.6)		1.43 (1.40-1.45)	0.93 (0.90-0.95)	0.93 (0.90-0.96)
Ductal and lobular	51,984 (88.7)	6,644 (11.3)		1.27 (1.23-1.30)	1.07 (1.04-1.11)	1.06 (1.02-1.10)
Other	81,294 (89.7)	9,311 (10.3)		1.13 (1.11-1.16)	1.14 (1.10-1.18)	1.10 (1.05-1.14)
**AJCC stage group**						
I	353,254 (84.8)	63,284 (15.2)	<.001	2.24 (2.18-2.30)	1.0 (reference)	1.0 (reference)
II	505,211 (92.2)	42,717 (7.8)		1.06 (1.03-1.09)	0.53 (0.52-0.53)	0.49 (0.48-0.50)
III	229,679 (95.0)	12,039 (5.0)		0.66 (0.64-0.68)	0.28 (0.27-0.29)	0.26 (0.25-0.27)
IV	83,623 (92.6)	6,681 (7.4)		1.0 (reference)	0.26 (0.25-0.27)	0.25 (0.24-0.26)
**Molecular subtype**						
HR+/ERBB2-	392,012 (86.2)	62,876 (13.8)	<.001	2.36 (2.31-2.41)	2.26 (2.21-2.32)	2.29 (2.23-2.35)
HR+/ERBB2+	147,645 (94.7)	8,324 (5.3)		0.83 (0.81-0.85)	0.70 (0.68-0.73)	0.71 (0.68-0.73)
HR-/ERBB2+	65,958 (95.4)	3,153 (4.6)		0.70 (0.68-0.73)	0.71 (0.68-0.74)	0.70 (0.67-0.74)
TNBC	174,962 (93.6)	11,893 (6.4)		1.0 (reference)	1.0 (reference)	1.0 (reference)
**Tumor grade**						
1	91,256 (80.7)	21,829 (19.3)	<.001	3.63 (3.57-3.70)	2.27 (2.22-2.33)	2.30 (2.24-2.37)
2	423,678 (88.4)	55,521 (11.6)		1.99 (1.96-2.02)	1.48 (1.45-1.50)	1.51 (1.48-1.54)
3	575,910 (93.8)	37,940 (6.2)		1.0 (reference)	1.0 (reference)	1.0 (reference)
**Year of initial diagnosis**				——	——	0.96 (0.96-0.96)

Abbreviations: No., number; SD, standard deviation; OR; odds ratio; AOR, adjusted odds ratio; AJCC, American Joint Committee on Cancer; HR, hormone receptors; ERBB2, human epidermal growth factor receptor 2; TNBC, triple-negative breast cancer.
[a] *P* values were computed using Student’s t or Pearson’s Chi-square tests.
[b] Adjusted for all variables presented in the table.
[c] Based on the 2016 American Community Survey data, spanning years 2012–2016 and adjusted for 2016 inflation.
[d] Measured by matching the state and county FIPS code of the patient recorded at the time of diagnosis against 2013 files published by the United States Department of Agriculture Economic Research Service.

© 2024 Freeman JQ et al. *JAMA Network Open.*

eTable 9. Distributions of Chemotherapy “Declined/Received” among Patients With Early-Stage, HR+/ERBB2- Breast Cancer Post-Surgery, by Multigene Assay Testing Result

	**Decision on chemotherapy** (N=428571)		
	**Received** (n=367316 [85.7%])	**Declined** (n=61255 [14.3%])	***P* value** [a]
	No. (row %)	No. (row %)	
**Oncotype DX** (Genomic Health thresholds; before 2018)			
Not tested	221107 (88.1)	29988 (11.9)	<.001
Low RS (0-17)	9863 (69.1)	4416 (30.9)	
Intermediate RS(18-30)	31732 (74.8)	10702 (25.2)	
High RS (31-100)	19010 (91.3)	1811 (8.7)	
**Oncotype DX** (TAILORx thresholds; on/after 2018)			
Not tested	81000 (89.0)	10000 (11.0)	<.001
Low-Intermediate RS (0-25)	8384 (75.5)	2721 (24.5)	
High RS (26-100)	18306 (87.2)	2688 (12.8)	
**MammaPrint**			
Not tested	313654 (84.8)	53851 (14.7)	<.001
Low risk	1785 (79.5)	459 (20.5)	
High risk	10123 (89.8)	1149 (10.2)	

Abbreviation: RS, recurrence score.
[a] *P* values were computed using Pearson’s Chi-square tests.

eTable 10. Associated Characteristics with Decision on Hormone Therapy in Patients With Stage I-IV, Hormone Receptor-Positive Breast Cancer

	**Decision on hormone therapy (N=1,893,339)**				**Reduced Model**	**Full Model**
**Variable**	**Received** (n=1,798,976 [95.0%]) No. (row %)	**Declined** (n=94,363 [5.0%]) No. (row %)	***P* value** [a]	**Crude OR (95% CI)**	**AOR (95% CI)**	**AOR (95% CI)** [b]
**Age at diagnosis (years)**, mean (SD)	61.5 (12.9)	66.7 (14.1)	<.001			
Per 10-year increase				1.37 (1.36-1.38)	1.37 (1.36-1.37)	1.44 (1.42-1.45)
**Sex**						
Male	16,206 (95.8)	708 (4.2)	<.001	1.0 (reference)	—	1.0 (reference)
Female	1,782,770 (95.0)	93,655 (5.0)		1.20 (1.11-1.30)	—	1.35 (1.22-1.49)
**Race/Ethnicity**						
White	1,444,596 (94.8)	79,909 (5.2)	<.001	1.0 (reference)	1.0 (reference)	1.0 (reference)
Black	166,915 (95.8)	7,357 (4.2)		0.80 (0.78-0.82)	0.88 (0.85-0.90)	0.86 (0.83-0.89)
Asian or Pacific Islander	65,082 (95.9)	2,753 (4.1)		0.76 (0.74-0.79)	0.86 (0.82-0.90)	0.81 (0.77-0.85)
Hispanic	94,248 (97.0)	2,928 (3.0)		0.56 (0.54-0.58)	0.68 (0.65-0.71)	0.66 (0.63-0.69)
American Indian, Alaska Native, or Other	28,135 (95.2)	1,416 (4.8)		0.91 (0.86-0.96)	1.05 (0.98-1.12)	1.04 (0.96-1.11)
**Type of health insurance**						
Uninsured	28,906 (95.3)	1,420 (4.7)	<.001	1.27 (1.20-1.34)	—	1.61 (1.49-1.73)
Private or managed care	918,139 (96.3)	35,603 (3.7)		1.0 (reference)	—	1.0 (reference)
Medicaid	103,273 (95.5)	4,855 (4.5)		1.21 (1.18-1.25)	—	1.44 (1.38-1.50)
Medicare	706,663 (93.3)	50,686 (6.7)		1.85 (1.82-1.88)	—	1.02 (0.99-1.04)
Other government or unknown	41,995 (95.9)	1,799 (4.1)		1.10 (1.05-1.16)	—	1.00 (0.93-1.07)
**Median household income quartiles** [c]						
<$40,227	228,986 (95.5)	10,830 (4.5)	<.001	0.90 (0.88-0.92)	—	0.87 (0.84-0.90)
$40,227–$50,353	313,471 (95.0)	16,434 (5.0)		1.00 (0.98-1.02)	—	0.95 (0.92-0.97)
$50,354–$63,332	368,086 (94.8)	20,236 (5.2)		1.05 (1.03-1.07)	—	1.00 (0.98-1.02)
≥$63,333	663,691 (95.0)	34,802 (5.0)		1.0 (reference)	—	1.0 (reference)
**Rural/urban area** [d]						
Metropolitan	1,519,662 (95.0)	80,025 (5.0)	.390	1.0 (reference)	—	1.0 (reference)
Urban	206,420 (95.1)	10,714 (4.9)		0.99 (0.97-1.01)	—	0.96 (0.93-0.99)
Rural	27,178 (95.0)	1,432 (5.0)		1.00 (0.95-1.06)	—	0.96 (0.89-1.03)

© 2024 Freeman JQ et al. *JAMA Network Open.*

**Type of cancer program**						
Community	122,254 (94.9)	6,580 (5.1)	<.001	1.18 (1.14-1.21)	—	1.07 (1.03-1.11)
Comprehensive community	719,166 (94.6)	41,432 (5.5)		1.26 (1.24-1.28)	—	1.19 (1.16-1.22)
Academic or research	511,853 (95.6)	23,434 (4.4)		1.0 (reference)	—	1.0 (reference)
Integrated network	371,173 (94.9)	20,019 (5.1)		1.18 (1.16-1.20)	—	1.13 (1.10-1.16)
**Charlson-Deyo comorbidity score**						
0	1,496,908 (95.1)	77,222 (4.9)	<.001	1.0 (reference)	1.0 (reference)	1.0 (reference)
1	228,756 (95.0)	12,157 (5.1)		1.03 (1.01-1.05)	0.90 (0.88-0.92)	0.89 (0.87-0.91)
≥2	73,312 (93.6)	4,984 (6.4)		1.32 (1.28-1.36)	1.03 (1.00-1.07)	1.03 (0.99-1.07)
**Histologic type**						
Ductal	1,370,914 (95.0)	72,579 (5.0)	<.001	1.0 (reference)	1.0 (reference)	1.0 (reference)
Lobular	221,669 (95.9)	9,597 (4.2)		0.82 (0.80-0.84)	0.80 (0.78-0.82)	0.81 (0.79-0.83)
Ductal and lobular	109,441 (95.8)	4,773 (4.2)		0.82 (0.80-0.85)	0.85 (0.82-0.88)	0.85 (0.81-0.88)
Other	96,952 (92.9)	7,414 (7.1)		1.44 (1.41-1.48)	1.43 (1.38-1.47)	1.41 (1.37-1.47)
**AJCC stage group**						
I	1,067,585 (94.0)	67,690 (6.0)	<.001	3.04 (2.89-3.20)	1.0 (reference)	1.0 (reference)
II	500,722 (96.1)	20,169 (3.9)		1.93 (1.83-2.04)	0.75 (0.74-0.77)	0.73 (0.71-0.74)
III	158,502 (96.9)	4,998 (3.1)		1.51 (1.43-1.60)	0.62 (0.60-0.64)	0.59 (0.56-0.61)
IV	72,167 (98.0)	1,506 (2.0)		1.0 (reference)	0.32 (0.30-0.34)	0.31 (0.29-0.34)
**ERBB2 status**						
Negative	1,185,515 (94.8)	65,070 (5.2)	<.001	1.0 (reference)	1.0 (reference)	1.0 (reference)
Positive	150,437 (95.8)	6,666 (4.2)		0.81 (0.79-0.83)	0.99 (0.97-1.02)	1.01 (0.98-1.05)
**Tumor grade**						
1	463,468 (93.6)	31,684 (6.4)	<.001	1.60 (1.56-1.63)	1.16 (1.13-1.19)	1.19 (1.16-1.22)
2	863,633 (95.5)	41,149 (4.6)		1.11 (1.09-1.13)	0.91 (0.89-0.93)	0.93 (0.91-0.96)
3	352 (95.9)	15,087 (4.1)		1.0 (reference)	1.0 (reference)	1.0 (reference)
**Year of initial diagnosis**				—	—	0.99 (0.99-0.996)

Abbreviations: No., number; SD, standard deviation; OR; odds ratio; AOR, adjusted odds ratio; AJCC, American Joint Committee on Cancer; HR, hormone receptors; ERBB2, human epidermal growth factor receptor 2.

[a] *P* values were computed using Student's t or Pearson's Chi-square tests.

[b] Adjusted for all variables presented in the table.

[c] Based on the 2016 American Community Survey data, spanning years 2012–2016 and adjusted for 2016 inflation.

[d] Measured by matching the state and county FIPS code of the patient recorded at the time of diagnosis against 2013 files published by the United States Department of Agriculture Economic Research Service.

© 2024 Freeman JQ et al. *JAMA Network Open.*

eTable 11. Associated Characteristics with Decision on Radiation Therapy in Patients With Stage I-III Breast Cancer

	**Decision on radiotherapy (N=1,635,916)**				**Reduced Model**	**Full Model**
**Variable**	**Received** (n=1,536,640 [93.9%]) No. (row %)	**Declined** (n=99,276 [6.1%]) No. (row %)	***P* value** [a]	**Crude OR (95% CI)**	**AOR (95% CI)**	**AOR (95% CI)** [b]
**Age at diagnosis (years)**, mean (SD)	60.1 (12.1)	69.0 (13.9)	<.001			
Per 10-year increase				1.88 (1.87-1.90)	1.91 (1.90-1.92)	2.08 (2.06-2.10)
**Sex**						
Male	8,524 (92.2)	717 (7.8)	<.001	1.0 (reference)	—	1.0 (reference)
Female	1,528,116 (93.9)	98,559 (6.1)		0.77 (0.71-0.83)	—	1.04 (0.94-1.16)
**Race/Ethnicity**						
White	1,208,318 (93.8)	80,328 (6.2)	<.001	1.0 (reference)	1.0 (reference)	1.0 (reference)
Black	166,688 (93.8)	11,023 (6.2)		0.99 (0.97-1.02)	1.16 (1.13-1.19)	1.05 (1.02-1.08)
Asian or Pacific Islander	53,667 (94.8)	2,934 (5.2)		0.82 (0.79-0.85)	1.06 (1.02-1.11)	1.01 (0.96-1.06)
Hispanic	83,312 (95.9)	3,553 (4.1)		0.64 (0.62-0.66)	0.86 (0.82-0.89)	0.74 (0.70-0.77)
American Indian, Alaska Native, or other	24,655 (94.5)	1,774 (5.5)		0.87 (0.83-0.93)	1.08 (1.02-1.16)	1.02 (0.94-1.09)
**Type of health insurance**						
Uninsured	26,164 (93.8)	1,718 (6.2)	<.001	1.90 (1.81-2.00)	—	1.97 (1.83-2.12)
Private or managed care	841,589 (96.7)	29,105 (3.3)		1.0 (reference)	—	1.0 (reference)
Medicaid	94,715 (94.0)	6,060 (6.0)		1.85 (1.80-1.90)	—	1.87 (1.79-1.94)
Medicare	536,907 (89.9)	60,549 (10.1)		3.26 (3.21-3.31)	—	1.09 (1.07-1.12)
Other government or unknown	37,265 (95.3)	1,844 (4.7)		1.43 (1.36-1.50)	—	1.09 (1.02-1.16)
**Median household income quartiles** [c]						
<$40,227	197,226 (93.3)	14,226 (6.7)	<.001	1.27 (1.24-1.29)	—	1.16 (1.13-1.20)
$40,227–$50,353	266,656 993.6)	18,349 (6.4)		1.21 (1.19-1.23)	—	1.11 (1.08-1.13)
$50,354–$63,332	314,999 (93.8)	20,905 (6.2)		1.17 (1.15-1.19)	—	1.09 (1.07-1.12)
≥$63,333	571,143 (94.6)	32,502 (5.4)		1.0 (reference)	—	1.0 (reference)
**Rural/urban area** [d]						
Metropolitan	1,307,452 (94.0)	83,463 (6.0)	<.001	1.0 (reference)	—	1.0 (reference)
Urban	169,870 (93.3)	12,138 (6.7)		1.12 (1.10-1.14)	—	1.01 (0.99-1.04)
Rural	21,446 (93.1)	1,581 (6.9)		1.15 (1.10-1.22)	—	1.05 (0.98-1.12)
**Type of cancer program**						

Community	106,963 (93.4)	7,624 (6.7)	<.001	1.16 (1.13-1.19)	——	0.92 (0.89-0.95)
Comprehensive community	618,469 (93.7)	41,526 (6.3)		1.09 (1.08-1.11)	——	1.00 (0.98-1.02)
Academic or research	425,395 (94.2)	26,149 (5.8)		1.0 (reference)	——	1.0 (reference)
Integrated network	315,954 (93.7)	21,200 (6.3)		1.09 (1.07-1.11)	——	1.03 (1.01-1.06)
**Charlson-Deyo comorbidity score**						
0	1,306,679 (94.4)	77,329 (5.6)	<.001	1.0 (reference)	1.0 (reference)	1.0 (reference)
1	179,774 (92.3)	14,942 (7.7)		1.40 (1.38-1.43)	1.06 (1.04-1.09)	1.06 (1.03-1.08)
≥2	50,187 (87.8)	7,005 (12.3)		2.36 (2.30-2.42)	1.45 (1.41-1.50)	1.38 (1.34-1.43)
**Histologic type**						
Ductal	1,220,088 (94.1)	76,404 (5.9)	<.001	1.0 (reference)	1.0 (reference)	1.0 (reference)
Lobular	147,650 (93.5)	10,272 (6.5)		1.11 (1.09-1.13)	0.88 (0.86-0.91)	0.87 (0.85-0.90)
Ductal and lobular	77,843 (94.5)	4,564 (5.5)		0.94 (0.91-0.97)	0.90 (0.87-0.93)	0.92 (0.88-0.96)
Other	91,059 (91.9)	8,036 (8.1)		1.41 (1.38-1.44)	1.23 (1.19-1.27)	1.24 (1.19-1.28)
**AJCC stage group**						
I	898,821 (93.9)	58,428 (6.1)	<.001	1.11 (1.09-1.13)	1.0 (reference)	1.0 (reference)
II	443,139 (93.8)	29,438 (6.2)		1.13 (1.11-1.16)	1.23 (1.21-1.25)	1.26 (1.23-1.28)
III	194,680 (94.5)	11,410 (5.5)		1.0 (reference)	1.18 (1.15-1.21)	1.20 (1.16-1.23)
**Molecular subtype**						
HR+/ERBB2-	800,295 (92.7)	62,785 (7.3)	<.001	1.41 (1.37-1.44)	1.32 (1.28-1.36)	1.36 (1.31-1.41)
HR+/ERBB2+	102,541 (94.1)	6,417 (5.9)		1.12 (1.08-1.16)	1.29 (1.24-1.34)	1.30 (1.25-1.36)
HR-/ERBB2+	38,979 (94.4)	2,319 (5.6)		1.07 (1.02-1.12)	1.21 (1.15-1.28)	1.23 (1.16-1.31)
TNBC	120,169 (94.7)	6,702 (5.3)		1.0 (reference)	1.0 (reference)	1.0 (reference)
**Tumor grade**						
1	353,460 (93.0)	26,447 (7.0)	<.001	1.39 (1.36-1.41)	1.17 (1.14-1.20)	1.21 (1.17-1.24)
2	648,874 (93.9)	42,500 (6.2)		1.22 (1.20-1.24)	1.02 (1.00-1.05)	1.04 (1.01-1.06)
3	439,331 (94.9)	23,659 (5.1)		1.0 (reference)	1.0 (reference)	1.0 (reference)
**Year of initial diagnosis**				——	——	1.05 (1.05-1.06)

Abbreviations: No., number; SD, standard deviation; OR; odds ratio; AOR, adjusted odds ratio; AJCC, American Joint Committee on Cancer; HR, hormone receptors; ERBB2, human epidermal growth factor receptor 2; TNBC, triple-negative breast cancer.

[a] *P* values were computed using Student's t or Pearson's Chi-square tests.

[b] Adjusted for all variables presented in the table.

[c] Based on the 2016 American Community Survey data, spanning years 2012–2016 and adjusted for 2016 inflation.

[d] Measured by matching the state and county FIPS code of the patient recorded at the time of diagnosis against 2013 files published by the United States Department of Agriculture Economic Research Service.

© 2024 Freeman JQ et al. *JAMA Network Open.*

eTable 12. Associated Characteristics with Decision on Surgery in Patients With Stage I-III Breast Cancer

	Decision on surgery (N=2,590,963)				Reduced Model	Full Model
**Variable**	**Received** (n=2,575,117 [99.4%]) No. (row %)	**Declined** (n=15,846 [0.6%]) No. (row %)	***P* value** [a]	**Crude OR (95% CI)**	**AOR (95% CI)**	**AOR (95% CI)** [b]
**Age at diagnosis (years)**, mean (SD)	61.3 (13.2)	74.2 (15.0)	<.001			
Per 10-year increase				2.34 (2.30-2.37)	2.43 (2.39-2.47)	2.83 (2.77-2.90)
**Sex**						
Male	22,859 (99.4)	141 (0.6)	.977	1.0 (reference)	——	1.0 (reference)
Female	2,552,258 (99.4)	15,705 (0.6)		0.998 (0.84-1.18)	——	2.02 (1.59-2.57)
**Race/Ethnicity**						
White	2,027,960 (99.4)	11,325 (0.6)	<.001	1.0 (reference)	1.0 (reference)	1.0 (reference)
Black	272,282 (98.9)	3,015 (1.1)		1.98 (1.90-2.06)	2.41 (2.29-2.54)	2.01 (1.89-2.14)
Asian or Pacific Islander	91,476 (99.4)	593 (0.6)		1.16 (1.07-1.27)	1.68 (1.52-1.85)	1.29 (1.15-1.44)
Hispanic	141,672 (99.6)	622 (0.4)		0.79 (0.73-0.85)	1.13 (1.02-1.24)	0.80 (0.71-0.89)
American Indian, Alaska Native, or other	41,727 (99.3)	291 (0.7)		1.25 (1.11-1.40)	1.78 (1.55-2.04)	1.47 (1.26-1.72)
**Type of health insurance**						
Uninsured	42,509 (98.8)	508 (1.2)	<.001	5.17 (4.70-5.68)	——	4.83 (4.22-5.51)
Private or managed care	1,321,257 (99.8)	3,055 (0.2)		1.0 (reference)	——	1.0 (reference)
Medicaid	151,451 (99.2)	1,293 (0.9)		3.69 (3.46-3.94)	——	3.19 (2.91-3.48)
Medicare	995,327 (98.9)	10,646 (1.1)		4.63 (4.44-4.82)	——	0.94 (0.89-1.00)
Other government or unknown	64,573 (99.5)	344 (0.5)		2.30 (2.06-2.58)	——	1.17 (1.00-1.38)
**Median household income quartiles** [c]						
<$40,227	345,057 (99.2)	2,655 (0.8)	<.001	1.48 (1.41-1.55)	——	1.14 (1.07-1.22)
$40,227–$50,353	454,096 (99.3)	3,038 (0.7)		1.28 (1.23-1.34)	——	1.13 (1.07-1.20)
$50,354–$63,332	527,852 (99.4)	3,193 (0.6)		1.16 (1.11-1.21)	——	1.06 (1.01-1.12)
≥$63,333	941,860 (99.5)	4,908 (0.5)		1.0 (reference)	——	1.0 (reference)
**Rural/urban area** [d]						
Metropolitan	2,180,549 (99.4)	13,844 (0.6)	<.001	1.0 (reference)	——	1.0 (reference)
Urban	288,969 (99.5)	1,587 (0.6)		0.87 (0.82-0.91)	——	0.83 (0.77-0.89)
Rural	37,613 (99.5)	180 (0.5)		0.75 (0.65-0.87)	——	0.68 (0.55-0.83)
**Type of cancer program**						

© 2024 Freeman JQ et al. *JAMA Network Open.*

Community	179,701 (99.3)	1,324 (0.7)	<.001	1.15 (1.08-1.22)	——	0.89 (0.82-0.97)
Comprehensive community	1,029,939 (99.4)	6,304 (0.6)		0.95 (0.92-0.99)	——	0.88 (0.83-0.92)
Academic or research	711,786 (99.4)	4,571 (0.6)		1.0 (reference)	——	1.0 (reference)
Integrated network	530,036 (99.4)	3,323 (0.6)		0.98 (0.93-1.02)	——	0.90 (0.85-0.96)
**Charlson-Deyo comorbidity score**						
0	2,149,328 (99..4)	12,464 (0.6)	<.001	1.0 (reference)	1.0 (reference)	1.0 (reference)
1	324,786 (99.4)	2,025 (0.6)		1.08 (1.03-1.13)	0.72 (0.68-0.76)	0.75 (0.70-0.80)
≥2	101,003 (98.7)	1,357 (1.3)		2.32 (2.19-2.45)	1.15 (1.07-1.23)	1.03 (0.96-1.11)
**Histologic type**						
Ductal	2,024,200 (99.4)	11,373 (0.6)	<.001	1.0 (reference)	1.0 (reference)	1.0 (reference)
Lobular	257,696 (99.3)	1,824 (0.7)		1.26 (1.20-1.32)	0.83 (0.79-0.89)	0.84 (0.79-0.90)
Ductal and lobular	134,351 (99.6)	567 (0.4)		0.75 (0.69-0.82)	0.66 (0.60-0.73)	0.75 (0.67-0.83)
Other	158,870 (98.7)	2,082 (1.3)		2.33 (2.23-2.44)	0.98 (0.91-1.06)	1.00 (0.92-1.09)
**AJCC stage group**						
I	1,532,818 (99.6)	6,124 (0.4)	<.001	1.0 (reference)	1.0 (reference)	1.0 (reference)
II	781,682 (99.2)	6,305 (0.8)		2.02 (1.95-2.09)	2.42 (2.32-2.54)	2.69 (2.56-2.83)
III	260,617 (98.7)	3,417 (1.3)		3.28 (3.15-3.42)	4.11 (3.89-4.35)	4.32 (4.06-4.61)
**Molecular subtype**						
HR+/ERBB2-	1,308,031 (99.3)	9,245 (0.7)	<.001	1.39 (1.30-1.48)	1.55 (1.44-1.68)	1.67 (1.54-1.81)
HR+/ERBB2+	170,210 (99.3)	1,172 (0.7)		1.35 (1.24-1.47)	1.75 (1.59-1.91)	1.76 (1.59-1.95)
HR-/ERBB2+	69,341 (99.3)	481 (0.7)		1.36 (1.22-1.52)	1.53 (1.36-1.71)	1.54 (1.35-1.75)
TNBC	200,229 (99.5)	1,021 (0.5)		1.0 (reference)	1.0 (reference)	1.0 (reference)
**Tumor grade**						
1	585,439 (99.5)	3,076 (0.5)	<.001	1.16 (1.12-1.22)	1.30 (1.22-1.39)	1.33 (1.24-1.43)
2	1,087,047 (99.4)	6,653 (0.6)		0.94 (0.90-0.99)	1.28 (1.21-1.35)	1.27 (1.20-1.34)
3	732,995 (99.5)	3,624 (0.5)		1.0 (reference)	1.0 (reference)	1.0 (reference)
**Year of initial diagnosis**				——	——	1.12 (1.11-1.13)

Abbreviations: No., number; SD, standard deviation; OR; odds ratio; AOR, adjusted odds ratio; AJCC, American Joint Committee on Cancer; HR, hormone receptors; ERBB2, human epidermal growth factor receptor 2; TNBC, triple-negative breast cancer.

[a] *P* values were computed using Student's t or Pearson's Chi-square tests.

[b] Adjusted for all variables presented in the table.

[c] Based on the 2016 American Community Survey data, spanning years 2012–2016 and adjusted for 2016 inflation.

[d] Measured by matching the state and county FIPS code of the patient recorded at the time of diagnosis against 2013 files published by the United States Department of Agriculture Economic Research Service.

© 2024 Freeman JQ et al. *JAMA Network Open.*

eTable 13. Kaplan-Meier Estimates of Median Overall Survival Time in Breast Cancer Patients Stratified By Treatment Decision and Race/Ethnicity

Cohort	Treatment decision	Race/Ethnicity	Median follow-up time in months (IQR)	No. of subjects	No. of events	Median survival time in months [a] (95% CI)	*P* value [b]
**Chemotherapy cohort [c]**	**Among patents who declined treatment**		59.7 (31.7-99.0)				
		White		94,056	32,007	142.4 (140.7-144.0)	<.001
		Black		13,924	4,779	139.3 (133.3-147.6)	
		API		4,223	785	213.1 (202.4-.)	
		Hispanic		4,652	1,024	NR	
		AIANO		1,821	514	163.4 (152.4-188.4)	
	**Among patients who received treatment**		73.4 (39.4-119.7)				
		White		808,992	173,026	217.0 (215.8-.)	<.001
		Black		156,734	42,382	216.2 (210.9-.)	
		API		42,769	5,479	NR	
		Hispanic		74,707	12,385	NR	
		AIANO		18,781	3,272	NR	
**Hormone therapy cohort [d]**	**Among patents who declined treatment**		59.5 (33.5-96.8)				
		White		74,779	19,842	155.7 (153.0-158.4)	<.001
		Black		6,909	1,937	154.1 (141.5-159.8)	
		API		2,587	374	NR (202.4-.)	
		Hispanic		2,738	492	NR	
		AIANO		1,300	243	NR	
	**Among patients who received treatment**		70.4 (40.2-111.5)				
		White		1,347,194	252,339	212.4 (211.3-213.7)	<.001
		Black		154,328	33,472	208 (201.8-210.9)	
		API		59,455	5,503	NR (213.1-.)	
		Hispanic		86,435	10,299	NR	
		AIANO		25,853	3,666	NR	

© 2024 Freeman JQ et al. *JAMA Network Open.*

**Radiotherapy cohort** [e]	**Among patents who declined treatment**		50.8 (29.4-82.6)				
		White		74,178	23,492	119.6 (118.2-121.2)	<.001
		Black		10,199	3,256	123.3 (119.3-130.5)	
		API		2,709	464	213.1 (174.4-.)	
		Hispanic		3,274	681	173.9 (161.7-.)	
		AIANO		1,316	332	143.1 (134.8-157.1)	
	**Among patients who received treatment**		75.9 (43.5-119.9)				
		White		1,133,156	186,822	215.8 (215.0-216.2)	<.001
		Black		155,200	30,008	NR	
		API		49,361	4,326	NR	
		Hispanic		76,685	8,634	NR	
		AIANO		22,786	2,826	NR	
**Surgery cohort** [e]	**Among patents who declined treatment**		31.5 (15.0-54.8)				
		White		10,332	6,014	44.7 (43.5-46.1)	<.001
		Black		2,789	1,410	54.3 (51.4-58.3)	
		API		543	171	97.9 (85.8-123.3)	
		Hispanic		581	235	69.7 (61.3-81.0)	
		AIANO		258	130	60.6 (45.6-68.7)	
	**Among patients who received treatment**		73.2 (41.1-116.6)				
		White		1,905,253	378,074	212.4 (211.4-213.4)	<.001
		Black		253,982	55,874	212.6 (209.9-.)	
		API		84,204	8,052	NR	
		Hispanic		130,993	16,647	NR	
		AIANO		38,594	5,575	NR	

Abbreviations: IQR, interquartile range; No., number; CI, confidence interval; NR=not reached; API, Asian or Pacific Islander; AIANO, American Indian, Alaska Native, or Other.
[a] Median survival time and 95% CIs were estimated using the Kaplan-Meier method.
[b] *P* value was calculated using the log-rank test.
[c] among patients with stage I-IV breast cancer.

© 2024 Freeman JQ et al. *JAMA Network Open.*

[d] among patients with stage I-IV, hormone receptor-positive breast cancer.
[e] among patients with stage I-III breast cancer.

© 2024 Freeman JQ et al. *JAMA Network Open.*

eTable 14. Kaplan-Meier Estimated 5-Year and 10-Year Overall Survival of Breast Cancer Patients Stratified by Treatment Decision

	**Among patients who declined treatment**		**Among patients who received treatment**	
	**5-Year OS (95% CI)** [a]	**10-Year OS (95% CI)** [a]	**5-Year OS (95% CI)** [a]	**10-Year OS (95% CI)** [a]
**Chemotherapy cohort** [b]				
**Race/Ethnicity**				
White	74.8 (74.5-75.1)	56.0 (55.6-56.4)	85.1 (85.0-85.2)	74.2 (74.1-74.3)
Black	70.8 (70.0-71.6)	54.5 (53.3-55.6)	77.7 (77.5-77.9)	66.3 (66.0-66.6)
Asian or Pacific Islander	84.6 (83.3-85.6)	71.6 (70.0-73.6)	90.0 (89.7-90.3)	82.6 (82.1-83.0)
Hispanic	81.4 (80.1-82.6)	66.3 (64.2-68.3)	86.5 (86.2-86.7)	77.3 (76.9-77.7)
American Indian, Alaska Native, or Other	76.3 (74.0-78.4)	60.3 (57.0-63.4)	86.9 (86.3-87.4)	77.6 (76.8-78.4)
**Hormone therapy cohort** [c]				
**Race/Ethnicity**				
White	81.4 (81.1-81.7)	61.0 (60.4-61.5)	88.5 (88.4-88.5)	74.7 (74.6-74.8)
Black	77.3 (76.2-78.4)	57.8 (56.0-59.5)	84.2 (84.0-84.4)	69.4 (69.0-69.7)
Asian or Pacific Islander	88.1 (86.6-89.5)	74.5 (71.7-77.1)	93.6 (93.4-93.8)	85.3 (84.8-85.7)
Hispanic	85.1 (83.4-86.6)	68.2 (65.3-70.9)	91.4 (91.2-91.6)	80.6 (80.1-81.0)
American Indian, Alaska Native, or Other	84.1 (81.7-86.3)	70.5 (66.6-74.0)	90.6 (90.2-91.0)	79.3 (78.6-80.0)
**Radiotherapy cohort** [d]				
**Race/Ethnicity**				
White	74.0 (73.7-74.4)	49.8 (49.3-50.4)	91.1 (91.0-91.1)	78.8 (78.7-78.9)
Black	71.9 (70.9-72.9)	51.2 (49.7-52.7)	86.6 (86.4-86.8)	74.1 (73.8-74.3)
Asian or Pacific Islander	85.2 (83.5-86.7)	69.7 (66.7-72.4)	94.4 (94.2-94.6)	86.8 (86.4-87.2)
Hispanic	81.1 (79.4-82.6)	61.0 (57.9-64.0)	92.2 (91.9-92.4)	82.6 (82.2-83.0)
American Indian, Alaska Native, or Other	78.6 (75.8-81.1)	58.7 (54.3-62.8)	92.5 (92.1-92.9)	82.8 (82.1-83.4)
**Surgery cohort** [d]				
**Race/Ethnicity**				
White	38.5 (37.4-39.6)	16.3 (15.1-17.5)	88.2 (88.1-88.2)	74.3 (74.3-74.4)
Black	47.2 (45.0-49.4)	22.6 (20.0-25.3)	84.2 (84.1-84.4)	70.6 (70.3-70.8)
Asian or Pacific Islander	65.0 (59.7-69.7)	43.2 (35.1-50.9)	93.6 (93.4-93.8)	85.5 (85.2-85.9)
Hispanic	55.8 (50.7-60.5)	36.3 (29.8-42.9)	90.9 (90.7-91.0)	80.8 (80.5-81.1)

© 2024 Freeman JQ et al. *JAMA Network Open.*

American Indian, Alaska Native, or Other	50.8 (43.4-57.7)	20.7 (12.3-30.7)	90.9 (90.5-91.2)	80.0 (79.5-80.6)

Abbreviations: OS, overall survival; CI, confidence interval.
[a] Estimated 5-year and 10-year rates and 95% CIs were calculated using the Kaplan-Meier method.
[b] among patients with stage I-IV breast cancer.
[c] among patients with stage I-IV, hormone receptor-positive breast cancer.
[d] among patients with stage I-III breast cancer.

© 2024 Freeman JQ et al. *JAMA Network Open.*

eTable 15. Associated Factors with Overall Survival in Patients With Stage I-IV Breast Cancer by Treatment Decision on Chemotherapy

	Among patients who declined treatment			Among patients who received treatment		
**Variable**	**aHR (95% CI)**	**aHR (95% CI)**	**aHR (95% CI)[a]**	**aHR (95% CI)[a]**	**aHR (95% CI)[a]**	**aHR (95% CI)[b]**
**Age at diagnosis** (per 10-year increase)	1.81 (1.78-1.83)	1.52 (1.50-1.54)	1.59 (1.56-1.62)	1.43 (1.43-1.44)	1.29 (1.29-1.30)	1.31 (1.30-1.32)
**Sex**						
Male	1.0 (reference)	——	1.0 (reference)	1.0 (reference)	——	1.0 (reference)
Female	0.76 (069.-0.78)	——	0.98 (0.85-1.13)	0.75 (0.72-0.78)	——	0.82 (0.77-0.87)
**Race/Ethnicity**						
White	1.0 (reference)	1.0 (reference)	1.0 (reference)	1.0 (reference)	1.0 (reference)	1.0 (reference)
Black	1.25 (1.20-1.29)	1.13 (1.08-1.18)	1.07 (1.02-1.13)	1.33 (1.32-1.35)	1.26 (1.24-1.28)	1.15 (1.13-1.17)
Asian or Pacific Islander	0.73 (0.67-0.78)	0.66 (0.60-0.73)	0.68 (0.61-0.75)	0.74 (0.71-0.76)	0.71 (0.68-0.73)	0.73 (0.70-0.76)
Hispanic	0.80 (0.75-0.86)	0.75 (0.69-0.82)	0.70 (0.63-0.77)	0.81 (0.79-0.83)	0.89 (0.87-0.91)	0.77 (0.75-0.80)
American Indian, Alaska Native, or Other	1.05 (0.95-1.15)	0.90 (0.79-1.03)	0.86 (0.75-1.00)	0.89 (0.86-0.93)	0.89 (0.84-0.94)	0.88 (0.83-0.93)
**Type of health insurance**						
Uninsured	2.48 (2.30-2.68)	——	1.75 (1.56-1.97)	2.01 (1.96-2.06)	——	1.56 (1.50-1.62)
Private or managed care	1.0 (reference)	——	1.0 (reference)	1.0 (reference)	——	1.0 (reference)
Medicaid	2.09 (1.98-2.20)	——	1.54 (1.43-1.66)	1.88 (1.85-1.90)	——	1.48 (1.45-1.51)
Medicare	1.21 (1.18-1.25)	——	1.10 (1.05-1.15)	1.37 (1.35-1.38)	——	1.27 (1.25-1.30)
Other government or unknown	1.24 (1.14-1.34)	——	1.10 (0.97-1.26)	1.28 (1.25-1.32)	——	1.20 (1.15-1.25)
**Median household income quartiles[b]**						
<$40,227	1.22 (1.19-1.26)	——	1.20 (1.14-1.25)	1.33 (1.31-1.35)	——	1.29 (1.26-1.31)
$40,227–$50,353	1.15 (1.11-1.18)	——	1.12 (1.08-1.17)	1.23 (1.22-1.25)	——	1.20 (1.18-1.22)
$50,354–$63,332	1.10 (1.07-1.13)	——	1.09 (1.05-1.14)	1.14 (1.13-1.15)	——	1.12 (1.10-1.14)
≥$63,333	1.0 (reference)	——	1.0 (reference)	1.0 (reference)	——	1.0 (reference)
**Type of cancer program**						
Community	1.26 (1.21-1.32)	——	1.27 (1.19-1.35)	1.15 (1.13-1.17)	——	1.18 (1.15-1.21)
Comprehensive community	1.12 (1.09-1.15)	——	1.12 (1.07-1.16)	1.07 (1.06-1.08)	——	1.11 (1.10-1.13)
Academic or research	1.0 (reference)	——	1.0 (reference)	1.0 (reference)	——	1.0 (reference)
Integrated network	1.13 (1.09-1.17)	——	1.08 (1.03-1.13)	1.05 (1.04-1.06)	——	1.05 (1.03-1.07)
**Charlson-Deyo comorbidity score**						

© 2024 Freeman JQ et al. *JAMA Network Open.*

0	—	1.0 (reference)	1.0 (reference)	—	1.0 (reference)	1.0 (reference)
1	—	1.27 (1.23-1.32)	1.28 (1.23-1.33)	—	1.27 (1.25-1.30)	1.24 (1.22-1.26)
≥2	—	1.83 (1.74-1.92)	1.83 (1.73-1.93)	—	1.90 (1.85-1.94)	1.78 (1.73-1.83)
**Histologic type**						
Ductal	—	1.0 (reference)	1.0 (reference)	—	1.0 (reference)	1.0 (reference)
Lobular	—	0.92 (0.88-0.97)	0.95 (0.90-1.00)	—	1.09 (1.06-1.11)	1.11 (1.08-1.14)
Ductal and lobular	—	0.84 (0.78-0.90)	0.87 (0.80-0.93)	—	1.00 (0.97-1.03)	1.02 (0.99-1.06)
Other	—	1.22 (1.15-1.29)	1.20 (1.13-1.28)	—	1.35 (1.32-1.38)	1.35 (1.32-1.38)
**AJCC stage group**						
I	—	1.0 (reference)	1.0 (reference)	—	1.0 (reference)	1.0 (reference)
II	—	1.86 (1.79-1.92)	1.78 (1.72-1.85)	—	1.77 (1.74-1.81)	1.73 (1.69-1.76)
III	—	4.11 (3.94-4.30)	3.80 (3.63-3.99)	—	4.03 (3.95-4.11)	3.76 (3.68-3.85)
IV	—	15.78 (14.98-16.63)	14.82 (14.00-15.68)	—	15.76 (15.43-16.10)	14.74 (14.39-15.09)
**Molecular subtype**						
HR+/ERBB2-	—	0.62 (0.60-0.65)	0.63 (0.60-0.66)	—	0.60 (0.60-0.61)	0.61 (0.60-0.62)
HR+/ERBB2+	—	0.77 (0.73-0.82)	0.78 (0.73-0.82)	—	0.46 (0.45-0.47)	0.48 (0.47-0.49)
HR-/ERBB2+	—	0.98 (0.91-1.05)	0.98 (0.91-1.05)	—	0.52 (0.51-0.53)	0.54 (0.52-0.55)
TNBC	—	1.0 (reference)	1.0 (reference)	—	1.0 (reference)	1.0 (reference)
**Tumor grade**						
1	—	0.64 (0.61-0.67)	0.65 (0.61-0.68)	—	0.60 (0.59-0.62)	0.61 (0.59-0.63)
2	—	0.73 (0.71-0.76)	0.74 (0.71-0.77)	—	0.74 (0.73-0.75)	0.75 (0.74-0.76)
3	—	1.0 (reference)	1.0 (reference)	—	1.0 (reference)	1.0 (reference)
**Year of initial diagnosis**	—	—	1.00 (0.99-1.01)	—	—	0.99 (0.9-0.99)

Abbreviations: CI, confidence interval; aHR, adjusted hazard ratio; AJCC, American Joint Committee on Cancer; HR, hormone receptors; ERBB2, human epidermal growth factor receptor 2; TNBC, triple-negative breast cancer.

[a] Adjusted for all variables presented in the table.

[b] Based on the 2016 American Community Survey data, spanning years 2012–2016 and adjusted for 2016 inflation.

© 2024 Freeman JQ et al. *JAMA Network Open.*

eTable 16. Associated Factors with Overall Survival in Patients With Stage I-IV, HR-Positive Breast Cancer by Treatment Decision on Hormone Therapy

	Among patients who declined treatment			Among patients who received treatment		
**Variable**	**aHR (95% CI)**	**aHR (95% CI)**	**aHR (95% CI)[a]**	**aHR (95% CI)**	**aHR (95% CI)**	**aHR (95% CI)[a]**
**Age at diagnosis** (per 10-year increase)	1.87 (1.84-1.90)	1.79 (1.76-1.82)	1.90 (1.86-1.95)	1.92 (1.91-1.93)	1.82 (1.82-1.83)	1.90 (1.89-1.91)
**Sex**						
Male	1.0 (reference)	——	1.0 (reference)	1.0 (reference)	——	1.0 (reference)
Female	0.66 (0.58-0.76)	——	0.82 (0.69-0.99)	0.61 (0.59-0.63)	——	0.78 (0.74-0.81)
**Race/Ethnicity**						
White	1.0 (reference)	1.0 (reference)	1.0 (reference)	1.0 (reference)	1.0 (reference)	1.0 (reference)
Black	1.41 (1.34-1.49)	1.15 (1.08-1.23)	1.05 (0.97-1.13)	1.30 (1.28-1.32)	1.26 (1.24-1.28)	1.15 (1.13-1.17)
Asian or Pacific Islander	0.86 (0.77-0.96)	0.74 (0.65-0.85)	0.73 (0.63-0.84)	0.71 (0.69-0.73)	0.68 (0.66-0.70)	0.68 (0.66-0.71)
Hispanic	0.95 (0.86-1.05)	0.86 (0.77-0.97)	0.81 (0.71-0.92)	0.80 (0.78-0.82)	0.86 (0.82-0.91)	0.75 (0.73-0.77)
American Indian, Alaska Native, or Other	1.02 (0.89-1.17)	0.79 (0.66-0.95)	0.78 (0.64-0.95)	0.92 (0.88-0.95)	0.86 (0.82-0.91)	0.87 (0.82-0.91)
**Type of health insurance**						
Uninsured	2.39 (2.123-2.70)	——	1.71 (1.45-2.02)	2.18 (2.12-2.25)	——	1.72 (1.64-1.79)
Private or managed care	1.0 (reference)	——	1.0 (reference)	1.0 (reference)	——	1.0 (reference)
Medicaid	2.17 (2.02-2.34)	——	1.79 (1.62-1.98)	2.16 (2.12-2.20)	——	1.75 (1.70-1.79)
Medicare	1.11 (1.06-1.16)	——	1.04 (0.98-1.10)	1.20 (1.18-1.21)	——	1.16 (1.14-1.18)
Other government or unknown	1.20 (1.07-1.35)	——	1.11 (0.94-1.31)	1.21 (1.18-1.24)	——	1.22 (1.17-1.27)
**Median household income quartiles[b]**						
<$40,227	1.33 (1.28-1.39)	——	1.22 (1.15-1.29)	1.33 (1.31-1.34)	——	1.30 (1.28-1.32)
$40,227–$50,353	1.19 (1.15-1.24)	——	1.14 (1.09-1.21)	1.23 (1.22-1.25)	——	1.21 (1.19-1.23)
$50,354–$63,332	1.10 (1.06-1.14)	——	1.07 (1.02-1.21)	1.15 (1.13-1.16)	——	1.13 (1.11-1.15)
≥$63,333	1.0 (reference)	——	1.0 (reference)	1.0 (reference)	——	1.0 (reference)
**Type of cancer program**						
Community	1.31 (1.24-1.38)	——	1.19 (1.10-1.28)	1.19 (1.17-1.21)	——	1.21 (1.18-1.24)
Comprehensive community	1.11 (1.07-1.16)	——	1.07 (1.02-1.13)	1.08 (1.07-1.09)	——	1.12 (1.10-1.13)
Academic or research	1.0 (reference)	——	1.0 (reference)	1.0 (reference)	——	1.0 (reference)
Integrated network	1.11 (1.06-1.26)	——	1.03 (0.97-1.09)	1.07 (1.06-1.08)	——	1.07 (1.05-1.09)
**Charlson-Deyo comorbidity score**						
0	——	1.0 (reference)	1.0 (reference)	——	1.0 (reference)	1.0 (reference)
1	——	1.37 (1.31-1.43)	1.33 (1.27-1.41)	——	1.38 (1.36-1.40)	1.35 (1.33-1.37)

© 2024 Freeman JQ et al. *JAMA Network Open.*

≥2	——	2.10 (1.98-2.23)	2.07 (1.94-2.21)	——	2.29 (2.25-2.34)	2.20 (2.16-2.25)
**Histologic type**						
Ductal	——	1.0 (reference)	1.0 (reference)	——	1.0 (reference)	1.0 (reference)
Lobular	——	1.01 (0.95-1.07)	1.03 (0.97-1.10)	——	1.00 (0.98-1.01)	1.02 (1.01-1.04)
Ductal and lobular	——	0.87 (0.80-0.95)	0.89 (0.81-0.97)	——	0.92 (0.90-0.94)	0.95 (0.93-0.97)
Other	——	0.92 (0.86-0.99)	0.90 (0.84-0.97)	——	1.15 (1.13-1.18)	1.14 (1.12-1.17)
**AJCC stage group**						
I	——	1.0 (reference)	1.0 (reference)	——	1.0 (reference)	1.0 (reference)
II	——	1.95 (1.87-2.03)	1.85 (1.76-1.93)	——	1.63 (1.61-1.65)	1.60 (1.58-1.62)
III	——	4.48 (4.22-4.76)	4.21 (3.94-4.51)	——	3.28 (3.23-3.23)	3.11 (3.06-3.17)
IV	——	28.45 (25.95-31.19)	29.32 (26.53-32.41)	——	12.65 (12.45-12.86)	11.85 (11.64-12.06)
**ERBB2 status**						
Negative	——	1.0 (reference)	1.0 (reference)	——	1.0 (reference)	1.0 (reference)
Positive	——	1.06 (1.00-1.12)	1.07 (1.00-1.13)	——	0.86 (0.84-0.87)	0.87 (0.86-0.89)
**Tumor grade**						
1	——	0.57 (0.54-0.60)	0.57 (0.54-0.61)	——	0.63 (0.62-0.64)	0.65 (0.64-0.66)
2	——	0.71 (0.68-0.74)	0.72 (0.68-0.75)	——	0.72 (0.71-0.73)	0.73 (0.72-0.74)
3	——	1.0 (reference)	1.0 (reference)	——	1.0 (reference)	1.0 (reference)
**Year of initial diagnosis**	——	——	1.01 (1.00-1.01)	——	——	1.00 (1.00-1.01)

Abbreviations: CI, confidence interval; aHR, adjusted hazard ratio; AJCC, American Joint Committee on Cancer; HR, hormone receptors; ERBB2, human epidermal growth factor receptor 2.

[a] Adjusted for all variables presented in the table.

[b] Based on the 2016 American Community Survey data, spanning years 2012–2016 and adjusted for 2016 inflation.

© 2024 Freeman JQ et al. *JAMA Network Open.*

eTable 17. Associated Factors with Overall Survival in Patients With Stage I-III Breast Cancer by Treatment Decision on Radiotherapy

	Among patients who declined treatment			Among patients who received treatment		
**Variable**	**aHR (95% CI)**	**aHR (95% CI)**	**aHR (95% CI)[a]**	**aHR (95% CI)**	**aHR (95% CI)**	**aHR (95% CI)[a]**
**Age at diagnosis** (per 10-year increase)	1.71 (1.68-1.73)	1.64 (1.62-1.66)	1.72 (1.69-1.75)	1.70 (1.69-1.71)	1.60 (1.59-1.61)	1.66 (1.65-1.68)
**Sex**						
Male	1.0 (reference)	——	1.0 (reference)	1.0 (reference)	——	1.0 (reference)
Female	0.73 (0.64-0.83)	——	0.85 (0.72-1.01)	0.58 (0.56-0.61)	——	0.80 (0.74-0.85)
**Race/Ethnicity**						
White	1.0 (reference)	1.0 (reference)	1.0 (reference)	1.0 (reference)	1.0 (reference)	1.0 (reference)
Black	1.21 (1.16-1.26)	1.03 (0.98-1.09)	0.98 (0.92-1.04)	1.37 (1.35-1.39)	1.22 (1.20-1.24)	1.13 (1.11-1.16)
Asian or Pacific Islander	0.74 (0.67-0.82)	0.70 (0.62-0.78)	0.71 (0.62-0.80)	0.75 (0.73-0.78)	0.68 (0.65-0.71)	0.70 (0.66-0.73)
Hispanic	0.86 (0.79-0.93)	0.80 (0.72-0.88)	0.74 (0.66-0.82)	0.86 (0.84-0.88)	0.90 (0.87-0.93)	0.79 (0.76-0.81)
American Indian, Alaska Native, or Other	1.04 (0.92-1.16)	0.92 (0.79-1.07)	0.95 (0.81-1.12)	0.92 (0.88-0.95)	0.88 (0.84-0.94)	0.88 (0.83-0.94)
**Type of health insurance**						
Uninsured	1.83 (1.64-2.03)	——	1.50 (1.28-1.74)	1.81 (1.75-1.87)	——	1.55 (1.47-1.63)
Private or managed care	1.0 (reference)	——	1.0 (reference)	1.0 (reference)	——	1.0 (reference)
Medicaid	1.85 (1.74-1.97)	——	1.62 (1.49-1.76)	1.95 (1.91-1.98)	——	1.60 (1.56-1.65)
Medicare	1.08 (1.04-1.12)	——	1.07 (1.02-1.13)	1.25 (1.24-1.27)	——	1.22 (1.20-1.24)
Other government or unknown	1.15 (1.04-1.27)	——	1.04 (0.90-1.20)	1.18 (1.15-1.22)	——	1.17 (1.11-1.23)
**Median household income quartiles[b]**						
<$40,227	1.30 (1.25-1.35)	——	1.23 (1.17-1.30)	1.36 (1.34-1.38)	——	1.29 (1.27-1.32)
$40,227–$50,353	1.18 (1.14-1.22)	——	1.15 (1.10-1.20)	1.26 (1.24-1.27)	——	1.22 (1.20-1.24)
$50,354–$63,332	1.09 (1.05-1.13)	——	1.06 (1.02-1.11)	1.16 (1.15-1.18)	——	1.14 (1.12-1.16)
≥$63,333	1.0 (reference)	——	1.0 (reference)	1.0 (reference)	——	1.0 (reference)
**Type of cancer program**						
Community	1.39 (1.33-1.46)	——	1.28 (1.19-1.36)	1.21 (1.19-1.23)	——	1.22 (1.19-1.26)
Comprehensive community	1.20 (1.16-1.24)	——	1.16 (1.11-1.21)	1.11 (1.10-1.12)	——	1.13 (1.11-1.15)
Academic or research	1.0 (reference)	——	1.0 (reference)	1.0 (reference)	——	1.0 (reference)
Integrated network	1.18 (1.13-1.22)	——	1.10 (1.04-1.16)	1.09 (1.08-1.10)	——	1.07 (1.05-1.09)
**Charlson-Deyo comorbidity score**						
0	——	1.0 (reference)	1.0 (reference)	——	1.0 (reference)	1.0 (reference)
1	——	1.34 (1.30-1.40)	1.34 (1.28-1.40)	——	1.35 (1.33-1.37)	1.32 (1.30-1.35)

© 2024 Freeman JQ et al. *JAMA Network Open.*

≥2	——	1.94 (1.84-2.03)	1.96 (1.85-2.06)	——	2.21 (2.16-2.26)	2.12 (2.07-2.18)
**Histologic type**						
Ductal	——	1.0 (reference)	1.0 (reference)	——	1.0 (reference)	1.0 (reference)
Lobular	——	0.97 (0.92-1.02)	0.99 (0.94-1.05)	——	1.00 (0.98-1.03)	1.02 (1.00-1.04)
Ductal and lobular	——	0.90 (0.83-0.97)	0.93 (0.86-1.01)	——	0.98 (0.95-1.01)	1.00 (0.97-1.04)
Other	——	1.03 (0.97-1.09)	1.03 (0.96-1.10)	——	1.27 (1.24-1.30)	1.25 (1.22-1.29)
**AJCC stage group**						
I	——	1.0 (reference)	1.0 (reference)	——	1.0 (reference)	1.0 (reference)
II	——	1.74 (1.78-1.81)	1.67 (1.61-1.74)	——	1.76 (1.74-1.79)	1.72 (1.70-1.75)
III	——	3.27 (3.13-3.42)	3.08 (2.93-3.23)	——	3.89 (3.83-3.96)	3.69 (3.62-3.76)
**Molecular subtype**						
HR+/ERBB2-	——	0.64 (0.60-0.67)	0.64 (0.60-0.68)	——	0.63 (0.61-0.64)	0.63 (0.62-0.64)
HR+/ERBB2+	——	0.69 (0.64-0.74)	0.69 (0.64-0.74)	——	0.51 (0.50-0.52)	0.53 (0.51-0.54)
HR-/ERBB2+	——	0.70 (0.63-0.76)	0.70 (0.64-0.78)	——	0.56 (0.54-0.58)	0.58 (0.55-0.60)
TNBC	——	1.0 (reference)	1.0 (reference)	——	1.0 (reference)	1.0 (reference)
**Tumor grade**						
1	——	0.64 (0.61-0.67)	0.65 (0.62-0.69)	——	0.61 (0.60-0.63)	0.63 (0.61-0.64)
2	——	0.74 (0.71-0.77)	0.75 (0.72-0.78)	——	0.71 (0.70-0.72)	0.72 (0.71-0.74)
3	——	1.0 (reference)	1.0 (reference)	——	1.0 (reference)	1.0 (reference)
**Year of initial diagnosis**	——	——	1.00 (0.99-1.01)	——	——	0.99 (0.99-0.99)

Abbreviations: CI, confidence interval; aHR, adjusted hazard ratio; AJCC, American Joint Committee on Cancer; HR, hormone receptors; ERBB2, human epidermal growth factor receptor 2; TNBC, triple-negative breast cancer.

[a] Adjusted for all variables presented in the table.

[b] Based on the 2016 American Community Survey data, spanning years 2012–2016 and adjusted for 2016 inflation.

© 2024 Freeman JQ et al. *JAMA Network Open.*

eTable 18. Associated Factors with Overall Survival in Patients With Stage I-III Breast Cancer by Treatment Decision on Surgery

	**Among patients who declined treatment**			**Among patients who received treatment**		
**Variable**	**aHR (95% CI)**	**aHR (95% CI)**	**aHR (95% CI)[a]**	**aHR (95% CI)**	**aHR (95% CI)**	**aHR (95% CI)[a]**
**Age at diagnosis** (per 10-year increase)	1.36 (1.33-1.39)	1.36 (1.33-1.40)	1.41 (1.36-1.46)	1.87 (1.86-1.87)	1.79 (1.78-1.80)	1.86 (1.85-1.87)
**Sex**						
Male	1.0 (reference)	——	1.0 (reference)	1.0 (reference)	——	1.0 (reference)
Female	0.79 (0.63-0.995)	——	0.78 (0.56-1.08)	0.67 (0.65-0.68)	——	0.76 (0.73-0.79)
**Race/Ethnicity**						
White	1.0 (reference)	1.0 (reference)	1.0 (reference)	1.0 (reference)	1.0 (reference)	1.0 (reference)
Black	0.95 (0.88-1.01)	0.83 (0.77-0.90)	0.82 (0.75-0.91)	1.31 (1.30-1.33)	1.19 (1.17-1.21)	1.10 (1.09-1.12)
Asian or Pacific Islander	0.57 (0.48-0.68)	0.58 (0.47-0.71)	0.56 (0.44-0.71)	0.71 (0.69-0.73)	0.66 (0.64-0.68)	0.68 (0.66-0.71)
Hispanic	0.73 (0.64-0.85)	0.71 (0.60-0.84)	0.69 (0.57-0.84)	0.83 (0.81-0.84)	0.86 (0.84-0.88)	0.76 (0.74-0.78)
American Indian, Alaska Native, or Other	0.87 (0.72-1.06)	0.79 (0.63-0.99)	0.80 (0.62-1.03)	0.90 (0.87-0.92)	0.87 (0.83-0.90)	0.87 (0.83-0.91)
**Type of health insurance**						
Uninsured	1.30 (1.11-1.53)	——	0.96 (0.76-1.20)	1.80 (1.75-1.85)	——	1.58 (1.51-1.64)
Private or managed care	1.0 (reference)	——	1.0 (reference)	1.0 (reference)	——	1.0 (reference)
Medicaid	1.29 (1.15-1.45)	——	1.12 (0.96-1.31)	1.99 (1.96-2.02)	——	1.69 (1.65-1.73)
Medicare	1.10 (1.02-1.19)	——	0.99 (0.89-1.11)	1.20 (1.19-1.21)	——	1.17 (1.15-1.19)
Other government or unknown	1.00 (0.83-1.21)	——	0.72 (0.54-0.97)	1.16 (1.14-1.19)	——	1.18 (1.14-1.23)
**Median household income quartiles[b]**						
<$40,227	1.08 (1.01-1.16)	——	1.14 (1.03-1.25)	1.34 (1.33-1.36)	——	1.30 (1.29-1.32)
$40,227–$50,353	1.03 (0.96-1.10)	——	1.09 (1.00-1.19)	1.24 (1.23-1.25)	——	1.21 (1.20-1.23)
$50,354–$63,332	1.04 (0.97-1.10)	——	1.01 (0.92-1.10)	1.15 (1.14-1.16)	——	1.14 (1.12-1.15)
≥$63,333	1.0 (reference)	——	1.0 (reference)	1.0 (reference)	——	1.0 (reference)
**Type of cancer program**						
Community	1.27 (1.16-1.39)	——	1.27 (1.11-1.45)	1.25 (1.24-1.27)	——	1.23 (1.21-1.26)
Comprehensive community	1.22 (1.15-1.29)	——	1.27 (1.17-1.37)	1.14 (1.13-1.15)	——	1.14 (1.13-1.16)
Academic or research	1.0 (reference)	——	1.0 (reference)	1.0 (reference)	——	1.0 (reference)
Integrated network	1.18 (1.10-1.27)	——	1.25 (1.14-1.38)	1.12 (1.10-1.13)	——	1.09 (1.08-1.11)
**Charlson-Deyo comorbidity score**						
0	——	1.0 (reference)	1.0 (reference)	——	1.0 (reference)	1.0 (reference)
1	——	1.21 (1.11-1.31)	1.23 (1.12-1.35)	——	1.40 (1.38-1.41)	1.37 (1.35-1.39)
≥2	——	1.71 (1.55-1.88)	1.63 (1.47-1.81)	——	2.28 (2.24-2.32)	2.21 (2.18-2.25)

© 2024 Freeman JQ et al. *JAMA Network Open.*

**Histologic type**						
Ductal	—	1.0 (reference)	1.0 (reference)	—	1.0 (reference)	1.0 (reference)
Lobular	—	0.97 (0.88-1.07)	0.96 (0.86-1.06)	—	0.94 (0.93-0.96)	0.97 (0.95-0.98)
Ductal and lobular	—	0.94 (0.81-1.09)	1.00 (0.85-1.17)	—	0.93 (0.90-0.95)	0.95 (0.93-0.98)
Other	—	0.99 (0.89-1.11)	1.03 (0.92-1.16)	—	1.18 (1.16-1.20)	1.16 (1.13-1.18)
**AJCC stage group**						
I	—	1.0 (reference)	1.0 (reference)	—	1.0 (reference)	1.0 (reference)
II	—	1.31 (1.22-1.41)	1.28 (1.18-1.38)	—	1.67 (1.65-1.68)	1.62 (1.60-1.64)
III	—	1.75 (1.61-1.90)	1.72 (1.57-1.88)	—	3.55 (3.50-3.59)	3.33 (3.28-3.37)
**Molecular subtype**		—				
HR+/ERBB2-	—	0.50 (0.45-0.55)	0.48 (0.43-0.54)	—	0.66 (0.65-0.67)	0.66 (0.66-0.67)
HR+/ERBB2+	—	0.59 (0.52-0.67)	0.59 (0.51-0.68)	—	0.59 (0.58-0.60)	0.60 (0.59-0.62)
HR-/ERBB2+	—	0.61 (0.52-0.72)	0.57 (0.47-0.69)	—	0.62 (0.60-0.63)	0.64 (0.62-0.65)
TNBC	—	1.0 (reference)	1.0 (reference)	—	1.0 (reference)	1.0 (reference)
**Tumor grade**						
1	—	0.65 (0.59-0.72)	0.66 90.60-0.74)	—	0.65 (0.64-0.66)	0.66 (0.65-0.67)
2	—	0.77 (0.71-0.83)	0.78 (0.72-0.85)	—	0.72 (0.72-0.73)	0.74 (0.73-0.75)
3	—	1.0 (reference)	1.0 (reference)	—	1.0 (reference)	1.0 (reference)
**Year of initial diagnosis**	—	—	0.99 (0.98-1.00)	—	—	0.99 (0.99-0.99)

Abbreviations: CI, confidence interval; aHR, adjusted hazard ratio; AJCC, American Joint Committee on Cancer; HR, hormone receptors; ERBB2, human epidermal growth factor receptor 2; TNBC, triple-negative breast cancer.

[a] Adjusted for all variables presented in the table.

[b] Based on the 2016 American Community Survey data, spanning years 2012–2016 and adjusted for 2016 inflation.

eFigure. Kaplan-Meier Curves for Overall Survival Stratified by Race and Ethnicity in Patients Who Received Treatment

© 2024 Freeman JQ et al. *JAMA Network Open.*

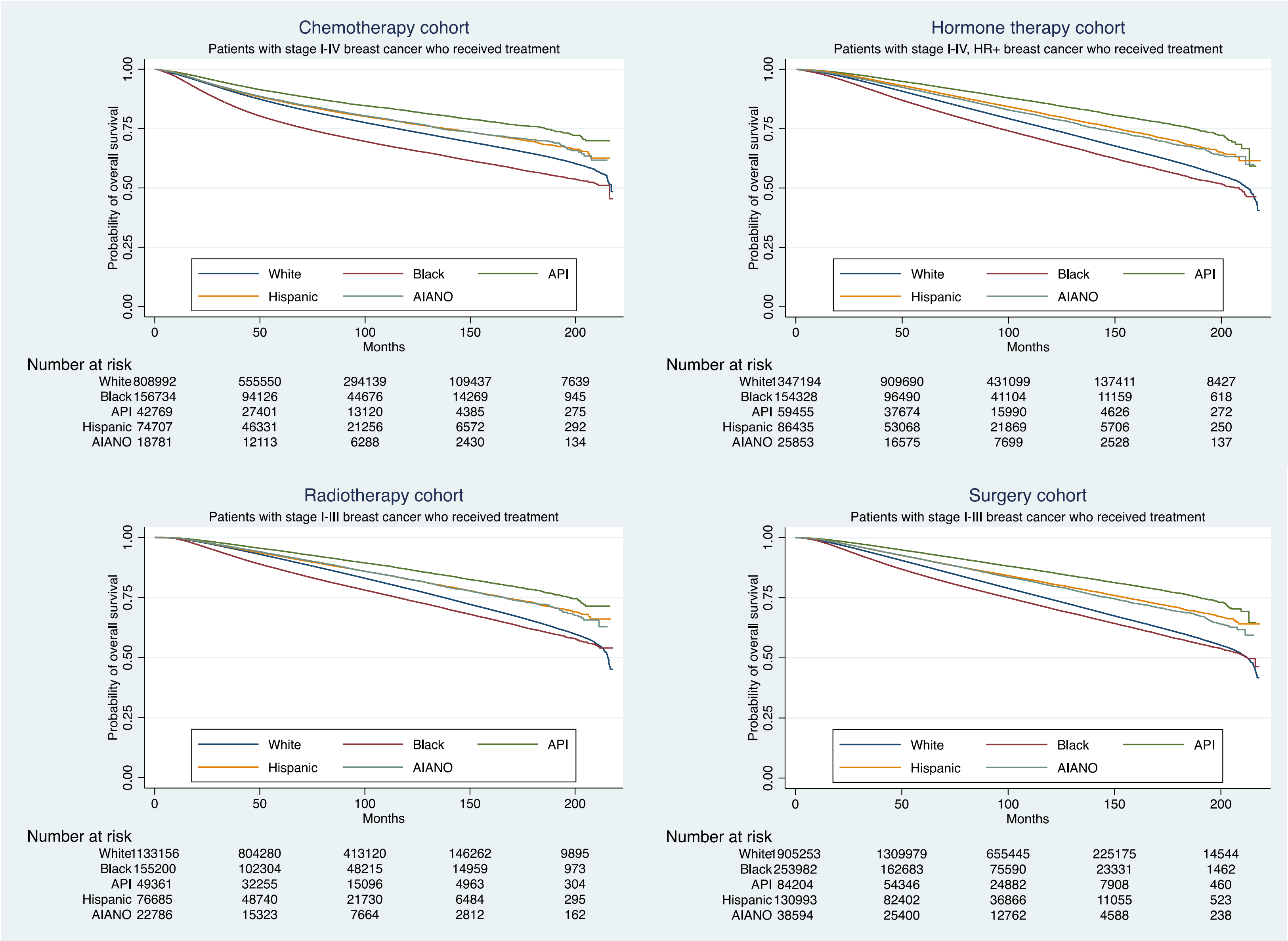

© 2024 Freeman JQ et al. *JAMA Network Open.*
