# Supplementary material for: Declination of Treatment, Racial and Ethnic Disparity, and Overall Survival in US Patients With Breast Cancer
Source: JAMA Netw Open. 2024 May 9;7(5):e249449. doi: 10.1001/jamanetworkopen.2024.9449 (PMC11082691; doi:10.1001/jamanetworkopen.2024.9449)
Supplement: Supplement 2. — Data Sharing Statement [file jamanetwopen-e249449-s002.pdf]

Data Sharing Statement

Freeman. Declination of Treatment, Racial and Ethnic Disparity, and Overall Survival in US Patients With Breast Cancer. *JAMA Netw Open*. Published May 09, 2024. doi:10.1001/jamanetworkopen.2024.9449

Data
Data available: No

Additional Information
Explanation for why data not available: Data types: De-identified patient data How to access data: Data for this analysis were obtained from the National Cancer Database (NCDB). Investigators associated with Commission on Cancer-accredited cancer programs can request the data by submitting a Participant User Data File (PUF) application to the American College of Surgeons via https://www.facs.org/quality-programs/cancer-programs/national-cancer-database. Supporting Documents Document types: None Additional Information Who can access the data: Anyone requesting the data Types of analyses: Any purpose Mechanisms of data availability: Requires a PUF application and a signed data use agreement according to the NCDB https://www.facs.org/quality-programs/cancer-programs/national-cancer-database/puf/
